# An investigation of the clinical impact and therapeutic relevance of a DNA damage immune response (DDIR) signature in patients with advanced gastroesophageal adenocarcinoma

**DOI:** 10.1016/j.esmoop.2024.103450

**Published:** 2024-05-13

**Authors:** M.A. Baxter, L.C. Spender, D. Cairns, S. Walsh, R. Oparka, R.J. Porter, S. Bray, G. Skinner, S. King, J. Turbitt, D. Collinson, Z.H. Miedzybrodzka, G. Jellema, G. Logan, R.D. Kennedy, R.C. Turkington, M.H. McLean, D. Swinson, H.I. Grabsch, S. Lord, M.J. Seymour, P.S. Hall, R.D. Petty

**Affiliations:** 1 Division of Molecular and Clinical Medicine, Ninewells Hospital and Medical School, University of Dundee, Dundee; 2 Tayside Cancer Centre, Ninewells Hospital and Medical School, NHS Tayside, Dundee; 3 Leeds Cancer Research UK Clinical Trials Unit, Leeds Institute of Clinical Trials Research, University of Leeds, Leeds; 4 Department of Pathology, Ninewells Hospital and Medical School, NHS Tayside, Dundee; 5 Department of Pathology, CRUK Scotland Centre, Institute of Genetics and Cancer, University of Edinburgh, Edinburgh; 6 Tayside Biorepository, University of Dundee, Dundee; 7 Genetics and Molecular Pathology Laboratory Services, NHS Grampian, Aberdeen; 8 School of Medicine, Medical Sciences, Nutrition and Dentistry, Polwarth Building, University of Aberdeen, Aberdeen; 9 Almac Diagnostic Services, Craigavon; 10 Patrick G Johnston Centre for Cancer Research, Queen’s University Belfast, Belfast; 11 St James’s University Hospital, Leeds Teaching Hospitals NHS Trust, Leeds, UK; 12 Department of Pathology, GROW School for Oncology and Reproduction, Maastricht University Medical Center, Maastricht, The Netherlands; 13 Division of Pathology and Data Analytics, Leeds Institute of Medical Research at St James’s University, University of Leeds, Leeds; 14 Department of Oncology, University of Oxford, Oxford; 15 Cancer Research UK Edinburgh Centre, MRC Institute of Genetics & Molecular Medicine, The University of Edinburgh, Western General Hospital, Crewe Road South, Edinburgh, UK

**Keywords:** gastroesophageal adenocarcinoma, DNA damage immune response, immune checkpoint inhibitors, epidermal growth factor receptor, tumour microenvironment

## Abstract

**Background:**

An improved understanding of which gastroesophageal adenocarcinoma (GOA) patients respond to both chemotherapy and immune checkpoint inhibitors (ICI) is needed. We investigated the predictive role and underlying biology of a 44-gene DNA damage immune response (DDIR) signature in patients with advanced GOA.

**Materials and methods:**

Transcriptional profiling was carried out on pretreatment tissue from 252 GOA patients treated with platinum-based chemotherapy (three dose levels) within the randomized phase III GO2 trial. Cross-validation was carried out in two independent GOA cohorts with transcriptional profiling, immune cell immunohistochemistry and epidermal growth factor receptor (*EGFR*) fluorescent *in situ* hybridization (FISH) (*n* = 430).

**Results:**

In the GO2 trial, DDIR-positive tumours had a greater radiological response (51.7% versus 28.5%, *P* = 0.022) and improved overall survival in a dose-dependent manner (*P* = 0.028). DDIR positivity was associated with a pretreatment inflamed tumour microenvironment (TME) and increased expression of biomarkers associated with ICI response such as *CD274* (programmed death-ligand 1, PD-L1) and a microsatellite instability RNA signature. Consensus pathway analysis identified EGFR as a potential key determinant of the DDIR signature. EGFR amplification was associated with DDIR negativity and an immune cold TME.

**Conclusions:**

Our results indicate the importance of the GOA TME in chemotherapy response, its relationship to DNA damage repair and EGFR as a targetable driver of an immune cold TME. Chemotherapy-sensitive inflamed GOAs could benefit from ICI delivered in combination with standard chemotherapy. Combining EGFR inhibitors and ICIs warrants further investigation in patients with EGFR-amplified tumours.

## Highlights

• The DDIR signature is predictive of chemotherapy response and dose-dependent survival in gastroesophageal adenocarcinoma.
• DDIR-positive tumours have an immune hot tumour microenvironment with expression of biomarkers of immunotherapy response.
• Epidermal growth factor receptor (EGFR) is identified as a potential driver of an immune cold tumour microenvironment.

## Introduction

Gastroesophageal cancer accounts for ∼1.3 million annual deaths globally.^1^ The majority of patients have advanced disease at diagnosis^2^ and median survival in unselected trial populations in this setting is less than a year.^3^ Although there are more biomarker-driven novel therapies being approved, including targeted therapies and immune checkpoint inhibitors (ICIs), cytotoxic chemotherapy remains an important part of clinical management and there is an ongoing need to identify biomarkers of treatment response.

The GO2 trial investigated chemotherapy dose de-escalation in an older and/or frailer population with advanced gastroesophageal cancer (ISRCTN44687907). In GO2, reduced dose doublet oxaliplatin/capecitabine (OX) chemotherapy (60% of standard dose of oxaliplatin 130mg/m2 on day 1, capecitabine 625mg/m2 on days 1-21, on a 21 day cycle) had non-inferior progression-free survival (PFS) and overall survival (OS) with improved patient experience compared with standard dose.^4^ The use of different chemotherapy doses provided a unique translational opportunity to investigate dose impact and potential biomarkers of response.

The DNA-damage immune response (DDIR) signature is a 44-gene transcriptional signature based on the loss of the Fanconi anaemia/BRCA (FA/BRCA) DNA-damage response pathway. Developed in breast cancer, the DDIR signature identifies patients who respond well to DNA-damaging neoadjuvant chemotherapy.^5^^,^^6^ The signature can be expressed as a continuous score or dichotomized into positive or negative. DDIR-positive tumours (exhibiting defective DNA damage repair) are characterized by an inflammatory tumour microenvironment (TME), up-regulation of interferon signalling genes, high lymphocytic infiltration,^7^^,^^8^ and enhanced signalling through the cGAS/STING pathway.^9^

In a subset of gastroesophageal adenocarcinoma (GOA) patients, chemotherapy promotes antitumour inflammation within the TME by reorganizing the T-cell compartment and inducing innate signalling pathways, including cGAS/STING in tumour cells, which is associated with chemotherapy response.^10^ Supporting this, DDIR-positive early-stage GOAs benefit more from neoadjuvant platinum-based chemotherapy with improved pathological response and survival.^7^ This has not been investigated in advanced-stage disease.

DNA-damaging chemotherapeutic agents, e.g. platinums, target vulnerabilities inherent in tumours with defective DNA damage repair machinery, leading to neoplastic cell death and improved outcomes for example in tumours with homologous recombination deficiency.^11^ Investigation in GOA indicates no association between homologous recombination deficiency and response to platinum-based chemotherapy, however, suggesting that defective DNA damage repair in tumours alone may have a limited impact on chemotherapy response in GOAs.^12^

Given these findings, and the emerging role of an inflamed TME in chemotherapy and ICI response, we hypothesized that the combination of defective DNA damage repair and an inflammatory TME, captured by the DDIR signature, could predict response and long-term survival to the DNA-damaging agent oxaliplatin within the GO2 trial population and provide further understanding of the biological basis of response to both chemotherapy and ICIs.

## Methods

This study was carried out according to the REporting recommendations for tumour MARKer prognostic studies (REMARK) (Supplementary Table 1, available at https://doi.org/10.1016/j.esmoop.2024.103450).^13^

### Patient samples

Formalin-fixed paraffin-embedded (FFPE) pre-chemotherapy tumour samples from 395 patients recruited to the GO2 trial^4^ were obtained. Samples were registered within NHS Tayside [Research Ethics Committee (REC) approval 17/ES/0130] and Grampian (REC 16/NS/0055) biorepositories. Only those with histologically confirmed adenocarcinoma and in whom RNA sequencing was successful were included in DDIR analysis. Radiological response was graded according to RECIST v1.1.^14^

For independent *in silico* validation, RNA sequencing from 306 oesophageal adenocarcinoma tumours was obtained from the Oesophageal Cancer Clinical and Molecular Stratification (OCCAMS) consortium. In addition, 124 pretreatment samples from patients with adenocarcinoma treated in NHS Grampian underwent *EGFR* FISH and immunohistochemistry (IHC) for CD8, CD4, FOXP3 and programmed death-ligand 1 (PD-L1).

### Gene expression profiling

Biopsies were reviewed for pathological subtype before marking for macrodissection and samples containing at least 10% adenocarcinoma tissue by area were taken forward. Where tumour material was limited, endoscopic biopsy fragments from the same patient were pooled. Methodology for RNA extraction and analysis was carried out as previously described.^7^ Further details can be found in the Supplementary Materials, available at https://doi.org/10.1016/j.esmoop.2024.103450.

### Microenvironment cell population analysis

The ‘*MCPcounter*’ (version MCPcounter_1.1.0) R package was downloaded from GitHub (https://github.com/ebecht/MCPcounter) and was used to generate microenvironment cell population (MCP) estimation scores for 10 stromal and immune cell infiltrates from the transcriptomic data of the cohorts.^15^ Estimates were compared between DDIR-positive and DDIR-negative to determine their stromal/immune content and the differences in cellular composition between the cancer types. MCP estimation scores were also generated according to *EGFR* FISH status.

### EGFR fluorescence in situ hybridisation

*EGFR* FISH was carried out and scored using an established protocol^16^ in NHS Grampian. Further details can be found in the Supplementary Materials, available at https://doi.org/10.1016/j.esmoop.2024.103450.

### IHC

IHC was carried out on tissue microarray (TMA) as previously described.^17^ Antibodies used are detailed in Supplementary Materials, available at https://doi.org/10.1016/j.esmoop.2024.103450. QuPath was carried out using published methodology^18^^,^^19^ and *QuPath Version 0.3.2.* Whole slide images (WSI) of immunostained TMA slides for CD4, CD8, FOXP3 and PD-L1 were imported. Further details can be found in the Supplementary Materials, available at https://doi.org/10.1016/j.esmoop.2024.103450. PD-L1 was scored manually by two independent observers, one of whom was an experienced gastrointestinal pathologist. Further details can be found in the Supplementary Materials, available at https://doi.org/10.1016/j.esmoop.2024.103450 and examples in Supplementary Figure S1, available at https://doi.org/10.1016/j.esmoop.2024.103450.

### Statistical analysis

Statistical analyses were conducted according to prespecified statistical analysis plans that were agreed upon before the inspection of any DDIR-stratified outcome data. Further details can be found in the Supplementary Materials and Supplementary Table 2, available at https://doi.org/10.1016/j.esmoop.2024.103450.

## Results

### GO2 trial advanced gastroesophageal cancer translational cohort

The GO2 trial (*n* = 559) demonstrated the non-inferiority of reduced dose chemotherapy in an older and/or frail population with advanced gastroesophageal cancer.^4^ From this patient cohort, RNA-sequencing data (and DDIR status) were obtained from 252 adenocarcinoma patients (Supplementary Methods, available at https://doi.org/10.1016/j.esmoop.2024.103450 and Supplementary Figure S2, available at https://doi.org/10.1016/j.esmoop.2024.103450).

A comparison of baseline characteristics demonstrated that the DDIR-analyzed cohort was representative of the whole adenocarcinoma trial population (*n* = 492) and a comparison between those patients with and without available RNA sequencing data revealed that there was no evidence of selection biases based on the GO2 stratification factors (Supplementary Table S3, available at https://doi.org/10.1016/j.esmoop.2024.103450) or difference in OS [hazard ratio (HR) 0.95, 95% confidence interval (CI) 0.76-1.17; *P* = 0.6] (Supplementary Figure S3, available at https://doi.org/10.1016/j.esmoop.2024.103450).

A total of 31/252 (12.3%) patients were classified as DDIR-positive, and the proportion of patients across the dose levels A, B and C was 33.7%, 29.8% and 36.5%, respectively. The DDIR-positive population was significantly older, as previously reported in the neoadjuvant setting, and frailer (Table 1).

**Table 1:** **Demographics of the GO2 adenocarcinoma population according to DDIR status**.

	DDIR-negative (*n* = 221)	DDIR-positive (*n* = 31)	*P* value
Age (years)			
Mean (SD)	75.0 (6.50)	78.4 (5.43)	**<0.001**
Median (Min, Max)	76.0 (52.0, 90.0)	80.0 (65.0, 87.0)	
Sex			
Male	166 (75.1%)	21 (67.7%)	0.51
Female	55 (24.9%)	10 (32.3%)	
ECOG PS			
0	30 (13.6%)	6 (19.4%)	0.346
1	121 (54.8%)	19 (61.3%)	
2+	69 (31.2%)	6 (19.4%)	
Missing	1 (0.5%)	0 (0%)	
Dose level			
100% OX (Level A)	74 (33.5%)	11 (35.5%)	0.876
80% OX (Level B)	67 (30.3%)	8 (25.8%)	
60% OX (Level C)	80 (36.2%)	12 (38.7%)	
Primary site			
Oesophagus	78 (35.3%)	8 (25.8%)	0.212
GOJ	65 (29.4%)	7 (22.6%)	
Gastric	78 (35.3%)	16 (51.6%)	
HER2 IHC status			
Positive	18 (8.1%)	4 (12.9%)	0.168
Negative	150 (67.9%)	24 (77.4%)	
Unavailable	53 (24.0%)	3 (9.7%)	
MMR IHC status			
Deficient	10 (4.5%)	3 (9.7%)	0.414
Proficient	157 (71.0%)	20 (64.5%)	
Unavailable	64 (29.0%)	8 (25.8%)	
Metastases present			
Yes	151 (68.3%)	19 (61.3%)	0.563
No	70 (31.7%)	12 (38.7%)	
GO2 frailty score			
Mean (SD)	2.75 (1.40)	2.94 (1.09)	**0.008**
Median (Min, Max)	3.00 (0, 8.00)	3.00 (1.00, 5.00)	
Missing	1 (0.5%)	0 (0%)	
GO2 frailty group			
Not frail	46 (20.8%)	2 (6.5%)	0.139
Slightly frail	52 (23.5%)	10 (32.3%)	
Severely frail	122 (55.2%)	19 (61.3%)	
Missing	1 (0.5%)	0 (0%)	

Bold indicates significant (*P*-value <0.05). GO2 Frailty Score and Group^4^ are defined by the number of geriatric domains with a deficit. Not frail = 0-1 domains, slightly frail = 2 domains, and severely frail = 3 or more domains. DDIR, DNA damage immune response; ECOG PS, Eastern Cooperative Oncology Group performance status; GOJ, gastroesophageal junction; HER2, human epidermal growth factor receptor 2; IHC, immunohistochemistry; MMR, mismatch repair; OX, oxaliplatin/capecitabine; SD, standard deviation.

### Outcomes according to DDIR status in the GO2 trial

Patients were followed up for a mandated 12 months after the commencement of systemic therapy.^4^ Radiological response and survival were analyzed in the 243 of the 252 patients who received chemotherapy.

A total of 21 (of 243; 8.6%) patients had no measurable disease on baseline scan and were excluded from response analysis (Supplementary Figure S4, available at https://doi.org/10.1016/j.esmoop.2024.103450). Progression or death before first scan was classed as progressive disease. The response rate was 31.5% (70/222) and the disease control rate was 66.7% (148/222). DDIR-positive patients had a significantly higher response rate than DDIR-negative patients; 51.7% versus 28.5% (*P* = 0.022) (Supplementary Figure S5, available at https://doi.org/10.1016/j.esmoop.2024.103450). Disease control rates were similar between the groups, 69.0% versus 66.3%, *P* = 0.944. There was no relationship between dose level and response rate (Supplementary Tables S4 and S5, available at https://doi.org/10.1016/j.esmoop.2024.103450).

**Figure 1:**
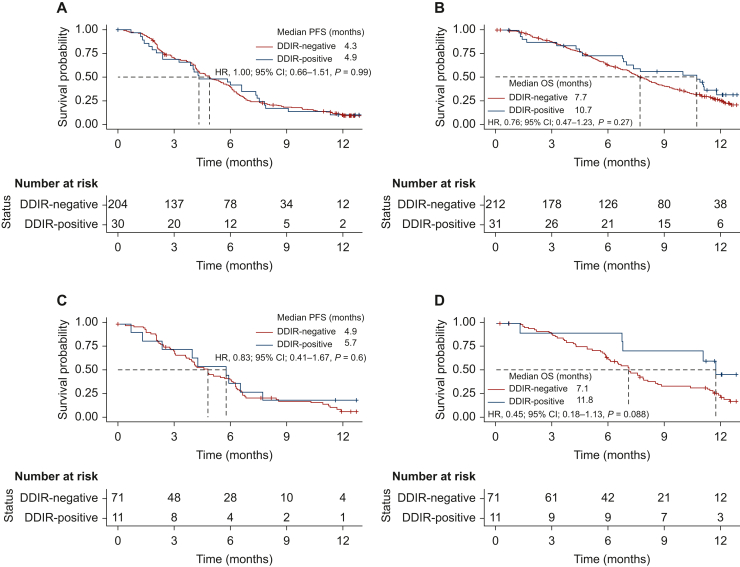
Kaplan–Meier curves stratified by DDIR status for (A) progression-free and (B) overall survival for 243 patients with advanced gastroesophageal adenocarcinoma treated with platinum-based chemotherapy in the GO2 trial and (C) progression-free and (D) overall survival for the subgroup of 82 patients treated with full dose platinum-based chemotherapy. CI, confidence interval; DDIR, DNA damage immune response; HR, hazard ratio; OS, overall survival; PFS, progression-free survival.

During follow-up, there were a total of 207 PFS and 182 OS events (Supplementary Figure S6, available at https://doi.org/10.1016/j.esmoop.2024.103450). No difference in PFS was observed between the DDIR groups; 4.9 months (95% CI 4.3-5.8 months) in DDIR-negative versus 4.3 months (95% CI 3.8-7.4 months) in DDIR-positive, (HR 1.00, 95% CI 0.66-1.51, *P* = 0.99) (Figure 1A).

There was also no significant difference in OS observed between the DDIR groups; 7.7 months (95% CI 7.1-8.6 months) in DDIR-negative versus 10.7 months in DDIR-positive (95% CI 7.0-not applicable), HR 0.76 (95% CI 0.47-1.23), *P* = 0.27 (Figure 1B). In Cox regression analysis, DDIR positivity had HRs for PFS and OS of 1.13 (95% CI 0.75-1.72), *P* = 0.56) (Supplementary Figure S7, available at https://doi.org/10.1016/j.esmoop.2024.103450) and 0.88 (95% CI 0.55-1.43, *P* = 0.62), respectively (Supplementary Figure S8A, available at https://doi.org/10.1016/j.esmoop.2024.103450).

In the DDIR-positive population, survival (Supplementary Figures S9 and S10, available at https://doi.org/10.1016/j.esmoop.2024.103450) and Cox regression analysis suggested an improved OS (but not PFS) with non-dose de-escalated chemotherapy; dose level C (60% OX) was associated with an HR of 4.35; 95% CI 1.18-16.1, *P* = 0.028 (Supplementary Figure S8B, available at https://doi.org/10.1016/j.esmoop.2024.103450). Importantly, there was no difference in the quality of life or the overall treatment utility [OTU; a composite clinical outcome measure of the effect of palliative treatments on individuals (7)] between dose levels (Supplementary Figure S11, available at https://doi.org/10.1016/j.esmoop.2024.103450 and Supplementary Table S6, available at https://doi.org/10.1016/j.esmoop.2024.103450).

Investigating all patients treated with dose level A (100% OX) (Supplementary Table S7, available at https://doi.org/10.1016/j.esmoop.2024.103450), although numerically longer, there was no significant improvement in OS in the DDIR-positive population—median OS 11.8 months (95% CI 6.8 months-not applicable) versus 7.1 months (95% CI 6.1-8.5 months) in DDIR-negative; HR 0.45 (95% CI 0.18-1.13), *P* = 0.088 (Figure 1D). There was also no significant difference in median PFS according to DDIR status (HR 0.83; 95% CI 0.41-1.67, *P* = 0.6) (Figure 1C).

In the DDIR-negative population, there was no dose level relationship with survival and also no difference in quality of life between dose levels (Supplementary Figures S12 and S13, available at https://doi.org/10.1016/j.esmoop.2024.103450), however, dose level A (100% OX) was associated with a poorer OTU (*P* < 0.001) (Supplementary Figure S14, available at https://doi.org/10.1016/j.esmoop.2024.103450).

Given the observed higher response rate, longer OS in DDIR-positive patients receiving dose level A (100% OX) and the known biology of the DDIR signature in other tumour types, we proceeded to investigate the association between DDIR score and immune/stromal composition, using gene expression profiles and MCP analysis.^15^

### The DDIR-positive TME reflects an immune-rich subtype

**Figure 2:**
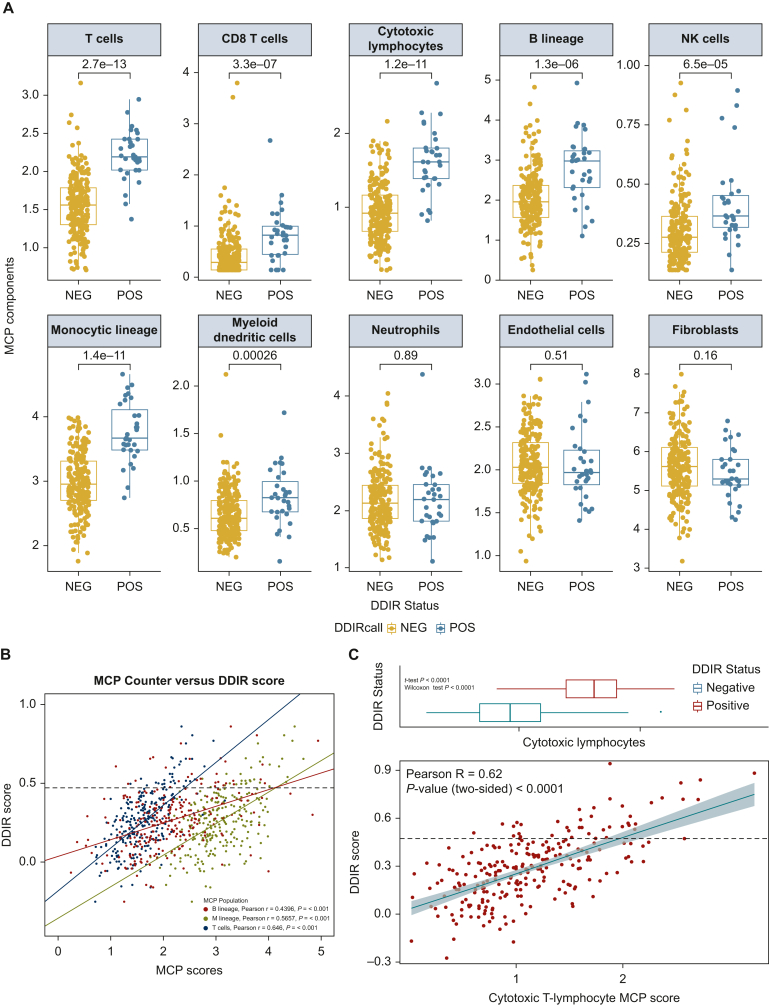
(A) Microenvironment cell population (MCP) scores of the individual immune cell types according to DDIR status. (B) Increased immune infiltrates correlated highly with DDIR positivity. MCP scores of three immune infiltrates—T-cells (blue), B lineage (red), and monocytic lineage (green)—correlated with DDIR scores with a line of best fit for each immune infiltrate. The black dashed horizontal line denotes the DDIR positivity cut-off. (C) Cytotoxic lymphocyte MCP scores correlated with DDIR scores in the GO2 population. The black horizontal line denotes the cut-off for DDIR positivity. Boxplot denotes the distribution of values for DDIR-positive and -negative status in the cohort. DDIR, DNA damage immune response; NEG, DDIR-negative; NK, natural killer; POS, DDIR-positive.

Using MCP analysis,^15^ we identified significant differences in several immune cell types according to DDIR status (Figure 2A). In addition, there were consistent correlations between DDIR scores and T-cell, B-cell, and monocytic immune lineages, confirming an increase in immune cell infiltration in DDIR-positive advanced GOA [Figure 2B. Pearson *r*; T cells = 0.646 (*P* < 0.001), B lineage = 0.4396 (*P* < 0.001), monocytic lineage = 0.5657 (*P* < 0.001)]. There was a strong correlation between the DDIR score and the cytotoxic T-lymphocyte score (Figure 2C).

**Figure 3:**
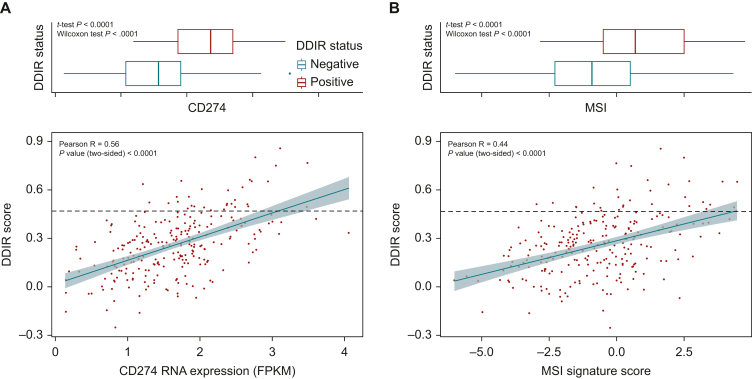
(A) Correlation of *CD274* RNA expression and DDIR Score in the GO2 GOA population. The black line denotes DDIR positivity. Boxplot denotes distribution of values for DDIR-positive and negative in the cohort. (B) Correlation of *MSI Signature* RNA expression score and DDIR Score in the GO2 GOA population. The black line denotes DDIR positivity. Boxplot denotes distribution of values for DDIR-positive and negative in the cohort. DDIR, DNA damage immune response; MSI, microsatellite instability.

Increasing DDIR score was also associated with an increase in *CD274* (*PD-L1*) expression and microsatellite instability (MSI) signature score^20^ (Figure 3A and B). The MSI signature was validated internally using IHC (Supplementary Figure S15, available at https://doi.org/10.1016/j.esmoop.2024.103450). DDIR-positive patients had significantly higher expression levels of both *CD274* and the MSI signature than DDIR-negative patients. Together, this supports the hypothesis that DDIR-positive advanced GOAs may also be more sensitive to ICI.

### Association between DDIR and Clara^T^ signature clusters

To extend this analysis of the underlying tumour biology of DDIR-positive GOAs, we carried out hierarchical clustering using the 92 individual gene signatures (of which DDIR is one), based on 10 hallmarks of cancer^21^ within the Almac Clara^T^ report. This identified six unique clusters (Supplementary Figure S16, available at https://doi.org/10.1016/j.esmoop.2024.103450 and Supplementary Table S8, available at https://doi.org/10.1016/j.esmoop.2024.103450). Cluster 2 had the highest proportion of DDIR-positive patients and the highest raw DDIR score (Supplementary Table S9, available at https://doi.org/10.1016/j.esmoop.2024.103450 and Supplementary Figure S17, available at https://doi.org/10.1016/j.esmoop.2024.103450). It was associated with homologous recombination deficits, cell cycle checkpoints and inflammatory and immune-oncology signatures. Analysis of the other 91 Clara^T^ signatures, considering those which predicted radiological response and a dose-dependent survival at a significance level of *P* < 0.05, identified the nuclear factor-kappa B (NFκB),^22^ T-cell inflamed GEP,^23^ TGCA CSF1 response^24^ and CTLA4 response^25^ signatures (Supplementary Table S10, available at https://doi.org/10.1016/j.esmoop.2024.103450).

Gene comparison analysis was carried out to identify shared genes between the significant predictive signatures (https://bioinformatics.psb.ugent.be/webtools/Venn). No genes were shared between all five signatures (Supplementary Table S11, available at https://doi.org/10.1016/j.esmoop.2024.103450); however, two genes, *CXCL10* and *IDO1,* were shared between three of the signatures. Both *CXCL10* and *IDO1* genes are significant contributors to the DDIR signature. *CXCL9* and *CXCL11*, the other CXCR3-related chemokines and *CCL5*, the other chemokine associated with the cGAS-STING pathway, were shared by two signatures.

**Figure 4:**
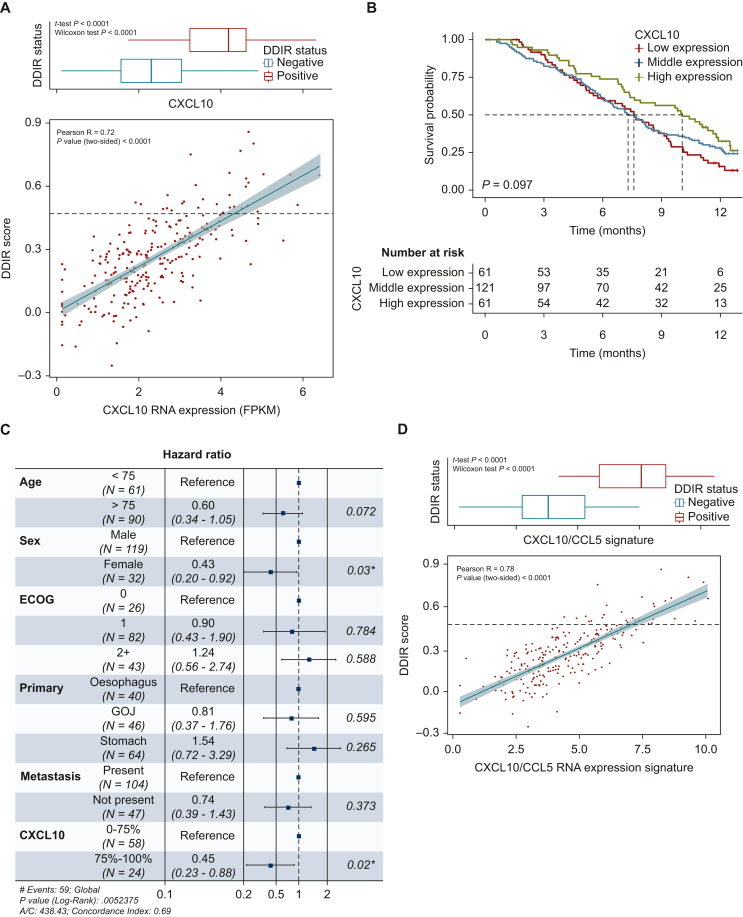
(A) Correlation between raw DDIR signature score and *CXCL10* RNA expression (FPKM) in the GO2 adenocarcinoma population. Boxplot denotes the distribution of expression values for the DDIR-positive and -negative samples in the cohort. (B) Overall survival in the GO2 adenocarcinoma population according to *CXCL10* RNA expression (FPKM). Low—bottom 25%, middle—25%–75%, high—top 25%. (C) Cox regression analysis for overall survival incorporating *CXCL10* RNA expression group. ∗indicates *P*-value <0.05. (D) Correlation between raw DDIR signature score and *CXCL10/CCL5* RNA expression (FPKM) signature in the GO2 adenocarcinoma population. Boxplot denotes distribution of values for DDIR-positive and -negative in the cohort. AIC, Akaike's Information Criterion; DDIR, DNA damage immune response; ECOG, Eastern Cooperative Oncology Group; GOJ, gastroesophageal junction.

As anticipated, there was a very good correlation between the DDIR score and *CXCL10* RNA expression (R = 0.72, *P* < 0.001) (Figure 4A). High RNA expression of *CXCL10* (defined as the top 25%) was associated with a survival advantage in the population as a whole (Figure 4B) and this benefit was maintained on Cox regression analysis; high expression of *CXCL10* was associated with an OS benefit in those who received dose level A (HR 0.45; *P* = 0.02) (Figure 4C).

The correlation between T-cell signatures and the DDIR signature, *CXCL10* expression alone or *CXCL10* in combination with *CCL5* or *IDO1*, was confirmed using The Cancer Genome Atlas (TCGA) publicly available repository. The combined *CXCL10/CCL5* signature performed as well as the DDIR signature in predicting T-cell signatures (Supplementary Table S12, available at https://doi.org/10.1016/j.esmoop.2024.103450). There was also a good correlation between this combined signature and the DDIR score (*R* = 0.78, *P* < 0.0001) in the GO2 population (Figure 4D) and high expression was associated with significantly higher TME infiltration of inflammatory immune cells on MCP analysis (Supplementary Figure S18, available at https://doi.org/10.1016/j.esmoop.2024.103450). The combined *CXCL10/CCL5* signature was also associated with improved OS and response rate (Supplementary Table S13, available at https://doi.org/10.1016/j.esmoop.2024.103450 and Supplementary Figure S19, available at https://doi.org/10.1016/j.esmoop.2024.103450).

Given the findings in the GO2 cohort, the association between CXCL10 (as a key determinant of DDIR status) and the TME was subsequently explored in transcriptomic data from an external cohort of 306 oesophageal and gastroesophageal junctional adenocarcinoma samples obtained from the OCCAMS consortium (Supplementary Table S14, available at https://doi.org/10.1016/j.esmoop.2024.103450). *CXCL10* gene expression was significantly correlated with cytotoxic T-lymphocyte abundance within the TME; Pearson *R* = 0.587, *P* < 0.001 (Supplementary Figure S20, available at https://doi.org/10.1016/j.esmoop.2024.103450).

### Consensus pathway analysis and EGFR

We have observed a relationship between the DDIR signature, ORR and survival in advanced GOA patients treated with platinum-based chemotherapy. There is also an association of the DDIR signature with increased immune infiltrate and biomarkers of response to ICIs. This may indicate the potential importance of the DDIR signature in predicting clinical outcomes for patients treated with chemotherapy and ICIs which form the basis of first-line standard-of-care treatments.

Therefore, we investigated the potential upstream targetable determinants of the DDIR signature using consensus pathway analysis (http://cpdb.molgen.mpg.de/). This analysis identified EGFR as a key hub (Supplementary Figure S21, available at https://doi.org/10.1016/j.esmoop.2024.103450). *EGFR* gene RNA expression had no association with ORR, PFS or OS in the GO2 cohort (Supplementary Figure S22, available at https://doi.org/10.1016/j.esmoop.2024.103450), however, it was negatively associated with DDIR score (Supplementary Figure S23, available at https://doi.org/10.1016/j.esmoop.2024.103450). None of the tumours with *EGFR* expression above 6 fragments per kilobase of tanscript per million mapped reads were DDIR-positive. EGFR-amplified GOAs are known to have higher *EGFR* RNA expression levels and are also known to benefit from treatment with EGFR inhibitors.^26^^,^^27^ This may therefore suggest that the DDIR-negative tumours with high *EGFR* RNA expression are EGFR-driven and targetable.

We proceeded to investigate the role of *EGFR* in relation to the DDIR signature and immune cell infiltrates in GOAs by measuring *EGFR* gene copy number using FISH testing, which is a predictive biomarker for EGFR inhibitors in GEAs.^16^

*EGFR* FISH was carried out on blindly stratified (based on baseline characteristics) GOA samples from the GO2 population (*n* = 143; 31 DDIR-positive and 112 DDIR-negative) (Supplementary Figure S24, available at https://doi.org/10.1016/j.esmoop.2024.103450). The selected samples were demographically similar to the GOA population as a whole (Supplementary Table S15, available at https://doi.org/10.1016/j.esmoop.2024.103450). Results were obtained for 124 (86.8%) samples. Of these, 30 (24.2%) were *EGFR* FISH-positive (defined as amplification or high polysomy) and 94 (75.8%) were *EGFR* FISH-negative (Supplementary Table S16, available at https://doi.org/10.1016/j.esmoop.2024.103450).^16^

Baseline demographics were well balanced (Supplementary Table S16, available at https://doi.org/10.1016/j.esmoop.2024.103450). Consistent with our previous findings, the *EGFR* FISH-positive cohort, and in particular *EGFR* amplification, had significantly lower DDIR scores and proportion of DDIR-positive patients (Supplementary Table S16, available at https://doi.org/10.1016/j.esmoop.2024.103450). There was no impact on OS according to FISH status (HR 1.34, *P* = 0.2).

*EGFR* FISH results according to DDIR status are shown in Supplementary Table S17, available at https://doi.org/10.1016/j.esmoop.2024.103450. Only 12% of *EGFR* FISH-positive were also DDIR-positive, contrasting with 27.3% of *EGFR* FISH-negative patients also being DDIR-positive, *P* = 0.183. Importantly, none of the DDIR-positive patients were *EGFR* amplified.

Next, we assessed *EGFR* FISH in relation to immune phenotype based on transcriptomic MCP analysis. On MCP analysis, tumours with *EGFR* amplification had a significantly lower abundance of cytotoxic T cells compared with FISH-negative tumours (*P* = 0.0036) (Supplementary Figures S25 and S26, available at https://doi.org/10.1016/j.esmoop.2024.103450).

#### Validation of EGFR status and immune cell phenotype by immunohistochemistry

To validate the association between *EGFR* amplification (by FISH) and immune cell infiltration (by IHC), 124 GOA FFPE pretreatment tumour specimens within a TMA were analyzed as previously published.^17^^,^^28^ The TMA was an external patient cohort obtained from NHS Grampian, Scotland. Two cores per patient, from the centre of the tumour, were taken. QuPath Image analysis^19^ was carried out on sections from the same tumour blocks to investigate CD8, CD4 and FOXP3 infiltration (Supplementary Figure S1, available at https://doi.org/10.1016/j.esmoop.2024.103450). These markers were selected due to the relationship between T cells and response to ICI.^29^^,^^30^ The sections were also scored manually for IHC PD-L1 combined positivity score (CPS).

The demographics of the TMA population are shown in Supplementary Table S18, available at https://doi.org/10.1016/j.esmoop.2024.103450. Within this population, 31 (25%) were *EGFR* FISH-positive; 9 (7.3%) were *EGFR* amplified. Like the findings in GO2, *EGFR* amplification was associated with an immune cold TME (Supplementary Table S19, available at https://doi.org/10.1016/j.esmoop.2024.103450).

A total of 103 samples were available for analysis of PD-L1 CPS; 87 (83.7%) samples had a score <1% (Supplementary Table S20, available at https://doi.org/10.1016/j.esmoop.2024.103450). *EGFR* FISH status was not significantly associated with PD-L1 CPS. PD-L1 CPS was associated with an immune hot TME (Supplementary Table S21, available at https://doi.org/10.1016/j.esmoop.2024.103450).

## Discussion

Advanced GOA is associated with a very poor prognosis. There is a need to identify biomarkers of response and the underlying biology. In this study, we present a molecular analysis in samples from a completed randomized clinical trial and an investigation of underlying biology relevant to both chemotherapy and immunotherapy with our findings being validated in independent patient cohorts.

We demonstrate that the 44-gene DDIR signature, which captures a combination of defective DNA damage repair mechanisms and an inflammatory TME, is associated with a higher response rate to platinum-based chemotherapy and improved OS. While the increased response rate was observed across all dose levels from the GO2 trial, improved survival, potentially via stimulation of immune surveillance, appears to require the non-de-escalated chemotherapy dose (level A, 100% OX). A higher DDIR signature score was also associated with an inflamed TME and, like triple-negative breast and colorectal cancer,^8^^,^^31^ increased expression of biomarkers of ICI response. In contrast, *EGFR* amplification was associated with the reduction of expression of the DDIR signature and an immune cold TME.

The DDIR signature has predictive value for response to DNA-damaging chemotherapy in breast cancer and oesophageal adenocarcinoma in the curative setting,^5^^,^^7^ but not in advanced colorectal cancer.^8^ Our investigation is the first to determine the interaction of chemotherapy dose with DDIR and, in doing so, has provided novel insights for the application of DDIR as a predictive biomarker as well as the underlying therapeutically relevant tumour biology.

DDIR positivity was observed in 12.3% of the GO2 cohort. This was lower than the observed 24% in the curative setting.^7^ It was also lower than the rates observed in triple-negative breast cancer (62%),^6^ ovarian cancer (30%)^32^ and colorectal cancer (19%-35%).^8^^,^^33^

The lower rates of DDIR positivity seen in the advanced gastroesophageal setting compared with the neoadjuvant setting may be due to the older/frailer patient population in GO2 (i.e. a changing disease biology with age). It may also reflect the impact of a differing biology across stage, which would support recent data in oesophageal adenocarcinoma, suggesting differences in mutational signatures with stage.^34^ Interestingly, advanced-stage colorectal cancer patients also have lower rates of DDIR positivity than in the localized setting.^33^

There may also be a contribution of a greater benefit of DDIR-positive tumours following DNA-damaging systemic therapy in the neoadjuvant/adjuvant setting.^7^ Accordingly, the DDIR phenotype impact on the TME may produce an initial improved response to neoadjuvant and adjuvant chemotherapy, but also results in longer-term disease control and immune surveillance.^7^ Additionally, previous platinum chemotherapy may alter the biology of the tumour or select out subgroups, for example, DDIR-negative that are resistant to chemotherapy.^35^ Supporting this concept, the DEBIOC study in oesophageal adenocarcinoma found that the post-neoadjuvant therapy DDIR signature score was significantly reduced.^36^

Within the GO2 cohort, DDIR-positive patients had a better response rate and a non-de-escalated dose of chemotherapy was associated with improved OS (HR, 0.23; 95% CI 0.06-0.85, *P* = 0.028). This improved OS occurred despite these patients being older and frailer (assessed by the GO2 frailty score), which has clinical relevance as reduced-dose chemotherapy is now widely adopted in this population. Of note, older adults (aged >75) had improved survival, independent of frailty, which may again indicate a different tumour biology according to age.

Importantly, the higher chemotherapy dose did not have a negative impact on patient experience or quality of life in the DDIR-positive population, suggesting that, overall, it was tolerated as well as the lower dose. This may be explained by the increased response with the higher dose, resulting in reduced tumour burden and improved symptom control. Together, these would improve treatment tolerance in the population. In addition, as toxicity reporting is capturing disease-related symptoms as well as treatment-related toxicity,^37^ the impact of the higher dose on experienced toxicity will be reduced.

The DNA damage-induced DDIR signature represents an inflamed baseline TME^5^^,^^9^ associated with increased T-cell, B-cell and monocytic immune lineages in both breast and colorectal cancer;^8^ this was tested in the GO2 population, which confirmed an increase in lymphocytic infiltration with an increasing DDIR score. This suggests that in this population, DNA-damaging chemotherapy induces a radiological response. For long-term disease control and thus improved survival, however, a higher dose of chemotherapy may be required to stimulate immune surveillance. This might explain the observed increased response rate to platinum-based chemotherapy in DDIR-positive GOAs across all dose levels from the GO2 trial, but improved survival only in those treated with non-de-escalated higher chemotherapy dose (level A, 100% OX).

Biologically, the DDIR effect in GOA, similar to breast cancer, appears to be driven by the chemokines CXCL10 and CCL5, the pro-inflammatory functions of which include T-cell recruitment and expansion.^38^ The relationship between CXCL10 and DDIR score is also observed in advanced colorectal cancer.^8^ The combined *CXCL10/CCL5* signature performed as well as the DDIR signature in predicting TCGA T-cell signatures. There was also a good correlation between this combined signature and the DDIR score, and it was also prognostic (both of response rate and survival). Together this suggests *CXCL10/CCL5* expression may warrant further investigation as a narrowed biomarker of DDIR status and chemosensitivity. A similar finding is observed in triple-negative breast cancer where *CXCL10* expression is related to a favourable prognosis.^39^ Importantly, *CXCL10* appears to be an important prognostic marker for response to ICIs,^40^, ^41^, ^42^ including in advanced oesophageal adenocarcinoma.^43^

As mentioned already, *CXCL10* expression is associated with improved response to ICI therapy. Other predictors of ICI response and improved outcome are PD-L1 and MSI, as well as the presence of tumour-infiltrating lymphocytes (TILs).^44^ In the GO2 population, both *PD-L1 (CD274)* and the MSI signature (which includes *CXCL10*) are expressed at significantly higher levels in DDIR-positive patients. *CXCL10* had a good correlation to both *PD-L1 (CD274)* and MSI signature scores, however, they appear to represent distinct populations. Therefore, it could be inferred that DDIR-positive patients are most likely to benefit from ICI therapy.

Overexpression of EGFR and gene CNG detected by FISH was associated with a less inflamed and immunologically colder TME. This may be a result of the known correlation between immune cold CIN tumours and *EGFR* amplification or a direct impact of EGFR signaling.^45^ Our consensus pathway analysis demonstrated that EGFR was a key hub and driver of the DDIR signature supporting a direct role for EGFR signalling.

The potential immunosuppressive role of EGFR is supported by evidence in other tumour groups. In breast cancer, EGFR positivity has been associated with increased FOXP3+ regulatory T cells,^46^ which are known to suppress antitumour immunity. In non-small-lung cancer, EGFR signalling, via interferon regulatory factor 1 (IRF1), reduces the expression of both CXCL10 and CCL5 while also increasing regulatory T-cell recruitment via CCL22.^47^ Therefore, the mechanism of an EGFR signalling-induced immune cold TME could be via alteration of the chemokine milieu.

Importantly, this process could potentially be counteracted by EGFR blockade which has been shown to promote the secretion of proinflammatory chemokines in both head and neck and breast cancer, as well as to improve responsiveness to anti-programmed cell death protein 1 (PD-1) blockade.^47^, ^48^, ^49^ This has not been investigated in advanced GOA and is the subject of ongoing research within our group.

The strengths of this study are that it is a clinical trial cohort and thus the clinical outcome data are reliable, and findings have been validated in independent cohorts. We present data from a large sample size which is unique in being from an older population which better represents the patients we see in clinical practice.

However, our study has several limitations. Firstly, there was an unexpectedly low prevalence of DDIR positivity, and therefore the survival findings should be interpreted with caution. In addition, the limited tumour tissue available on FFPE blocks resulted in reduced sample size for subsequent IHC analysis (e.g. HER2). The rates of PD-L1 positivity within the TMA were lower than expected; this may be a consequence of sampling during the creation of the TMA, with samples more likely to be taken from the centre of the tumour specimen or the result of age-related deglycosylation of the extracellular domain of PD-L1,^50^ and indicates that caution needs to be taken in the interpretation of these particular results.

We must also acknowledge that despite the DDIR signature containing several features which are known to be prognostic, it had no impact on PFS or OS in the population as whole. Added to this, the clinical significance of improved response rate can be questioned as it is not a good surrogate for the main outcomes of PFS and OS. The improvement in response rate, however, does imply increased sensitivity to chemotherapy, and the lack of clear survival benefit findings may reflect the impact of different dose levels, an underpowered study or the impact of the treatment on an older frailer cohort of patients. As such the clinical utility of the DDIR signature may be in fitter patients and also in giving an insight into underlying biology.

### Conclusions

In summary, our study shows that in advanced GOA, the DDIR signature can predict an improved response to oxaliplatin treatment. The OS benefit may require the standard, non-dose de-escalated chemotherapy regime. We have identified that the underlying biology of the DDIR signalling in GOA, similar to breast cancer, is associated with constitutive gene up-regulation of the chemokines *CXCL10* and *CCL5* and an inflamed TME. EGFR copy number gain and in particular amplification may have an inhibitory effect on this signalling; however, this needs further investigation.

This work also underscores the importance of the connection between DNA damage repair components and inflammation in the TME in determining GOA patient outcomes. Our data may provide rationale for the mechanistic investigation of the combination of ICI with EGFR inhibition in tumours with EGFR CNG as a means to enhance anticancer immune responses and improve the efficacy of immunotherapies.
