# Supplementary material for: An investigation of the clinical impact and therapeutic relevance of a DNA damage immune response (DDIR) signature in patients with advanced gastroesophageal adenocarcinoma
Source: ESMO Open. 2024 May 13;9(5):103450. doi: 10.1016/j.esmoop.2024.103450 (PMC11108838; doi:10.1016/j.esmoop.2024.103450)
Supplement: Supplementary Material [file mmc1.docx]

**Supplementary Methods/Data**

**The GO2 Trial**

The GO2 trial was selected for exploration of the role of dose de-escalation. The study was performed in accordance with the Declaration of Helsinki. All subjects provided written informed consent for biospecimen collection (REC Number 13/YH/0229). This was a translational sub-study of the GO2 trial funded by Cancer Research UK (C22029/A29637).

The GO2 trial is unique in that it recruited older and or frailer patients with advanced gastroesophageal cancer, felt to better represent real-world patients encountered in clinical practice[1]. The trial sought to investigate the role of chemotherapy dose de-escalation in this population. 559 patients were recruited and were randomised to either a ‘likely to benefit’ (n=514) or ‘uncertain to benefit’ (n=45) arm. In the ‘likely to benefit’ arm, patients were randomised to either 100% (Level A), 80% (Level B) or 60% (Level C) doublet chemotherapy regimen of oxaliplatin/capecitabine. 100% dose was oxaliplatin 130 mg/m^2^ on day 1 and capecitabine 625 mg/m^2^ twice daily on days 1-21, on a 21-day cycle. In the ‘uncertain to benefit’ arm, patients were randomised to either Level C or supportive care alone.

Patients were randomised using an automated telephone/web system and validated minimization algorithm, with age, ECOG PS, presence of metastases, histology, renal function, planned trastuzumab use, and centre as stratification factors. The treatment allocation was unblinded to both patients and clinicians. At baseline, each recruited patient had a detailed geriatric and quality-of-life assessment.

For the DDIR analysis, samples from patients were obtained from archival diagnostic tissue. The primary outcome for GO2 was PFS, but data were also available for overall survival (OS) and objective response rate (ORR).

**Patient and Public Involvement**

Patient and Public Involvement (PPI) relationships and structures, the views of oesophageal cancer patients and their representatives have been extensively considered during the development of the GO2 clinical trial from which samples and data for this project were derived. This included a review of potential future translational research that might be undertaken from bio-specimens collected.

**Supplementary Table 1:** Comparison of the reporting of the DDIR assay as a predictive marker in advanced gastroesophageal adenocarcinoma.

| **REMARK Guidelines Criteria** | **DDIR in advanced gastroesophageal adenocarcinoma** |
| --- | --- |
| \| [**INTRODUCTION**](https://www.ncbi.nlm.nih.gov/pmc/articles/PMC3362085/#s4) \| \| \| --- \| --- \| \| 1 \| State the marker examined, the study objectives, and any pre-specified hypotheses. \| \| [**MATERIALS AND METHODS**](https://www.ncbi.nlm.nih.gov/pmc/articles/PMC3362085/#s5) \| \| \| Patients \| \| \| 2 \| Describe the characteristics (for example, disease stage or co-morbidities) of the study patients, including their source and inclusion and exclusion criteria. \| \| 3 \| Describe treatments received and how chosen (for example, randomized or rule-based). \| \| Specimen characteristics \| \| \| 4 \| Describe type of biological material used (including control samples) and methods of preservation and storage. \| \| Assay methods \| \| \| 5 \| Specify the assay method used and provide (or reference) a detailed protocol, including specific reagents or kits used, quality control procedures, reproducibility assessments, quantitation methods, and scoring and reporting protocols. Specify whether and how assays were performed blinded to the study endpoint. \| \| Study design \| \| \| 6 \| State the method of case selection, including whether prospective or retrospective and whether stratification or matching (for example, by stage of disease or age) was used. Specify the time period from which cases were taken, the end of the follow-up period, and the median follow-up time. \| \| 7 \| Precisely define all clinical endpoints examined. \| \| 8 \| List all candidate variables initially examined or considered for inclusion in models. \| \| 9 \| Give rationale for sample size; if the study was designed to detect a specified effect size, give the target power and effect size. \| \| Statistical analysis methods \| \| \| 10 \| Specify all statistical methods, including details of any variable selection procedures and other model-building issues, how model assumptions were verified, and how missing data were handled. \| \| 11 \| Clarify how marker values were handled in the analyses; if relevant, describe methods used for cutpoint determination. \| \| [**RESULTS**](https://www.ncbi.nlm.nih.gov/pmc/articles/PMC3362085/#s6) \| \| \| Data \| \| \| 12 \| Describe the flow of patients through the study, including the number of patients included in each stage of the analysis (a diagram may be helpful) and reasons for dropout. Specifically, both overall and for each subgroup extensively examined report the number of patients and the number of events. \| \| 13 \| Report distributions of basic demographic characteristics (at least age and sex), standard (disease-specific) prognostic variables, and tumour marker, including numbers of missing values. \| \| Analysis and presentation \| \| \| 14 \| Show the relation of the marker to standard prognostic variables. \| \| 15 \| Present univariable analyses showing the relation between the marker and outcome, with the estimated effect (for example, hazard ratio and survival probability). Preferably provide similar analyses for all other variables being analyzed. For the effect of a tumor marker on a time-to-event outcome, a Kaplan-Meier plot is recommended. \| \| 16 \| For key multivariable analyses, report estimated effects (for example, hazard ratio) with confidence intervals for the marker and, at least for the final model, all other variables in the model. \| \| 17 \| Among reported results, provide estimated effects with confidence intervals from an analysis in which the marker and standard prognostic variables are included, regardless of their statistical significance. \| \| 18 \| If done, report results of further investigations, such as checking assumptions, sensitivity analyses, and internal validation. \| \| [**DISCUSSION**](https://www.ncbi.nlm.nih.gov/pmc/articles/PMC3362085/#s7) \| \| \| 19 \| Interpret the results in the context of the pre-specified hypotheses and other relevant studies; include a discussion of limitations of the study. \| \| 20 \| Discuss implications for future research and clinical value. \|   Note: we have changed ‘univariate’ to ‘univariable’ in item 15 for consistency with ‘multivariable’. | The marker examined was the DNA Damage Response Deficiency assay. We assessed the ability of a clinically validated DNA Damage Response Deficiency (DDIR) assay to predict prognosis following DNA damaging palliative chemotherapy in gastroesophageal adenocarcinoma.  See Methods and Figure 1. n=252 advanced GOA patients recruited to the GO2 trial treated with platinum-based chemotherapy. Validation: RNA-sequencing from n=306 patients with GOA adenocarcinoma from OCCAMS, EGFR FISH and QuPath analysis from n=124 patients with GOA from NHS Grampian.  FFPE tissue used for RNA-extraction from GO2 cohort and EGFR FISH analysis. Tissue microarray used for QuPath.  See Methods.  DNA Damage Response Deficiency Assay (see Methods section and Mulligan et al J Natl Cancer Inst. 2014 Jan;106(1)).  See Results and Methods.  See Results and Methods.  See Results and Methods.  See Supplementary data.  See Supplementary data.  See Supplementary data.  See Results, Figure 1 and Supplementary data  See Table 2 and Supplementary data  See Results and Supplementary data  See Results and Supplementary data  See Results and Supplementary data  See Results and Supplementary data  See Results and Supplementary data  See Discussion  See Discussion |

**Oesophageal Cancer Clinical and Molecular Stratification (OCCAMS) Consortium**

Rebecca C. Fitzgerald^1^, Paul A.W. Edwards^1,2^, Nicola Grehan^1,5^, Barbara Nutzinger^1^, Aisling M Redmond^1^, Christine Loreno^1^, Sujath Abbas^1^, Adam Freeman^1^ Elizabeth C. Smyth^5^, Maria O’Donovan^1,3^, Ahmad Miremadi^1,3^, Shalini Malhotra^1,3^, Monika Tripathi^1,3^, Calvin Cheah^1^, Hannah Coles^1^ Curtis Millington^1^, Matthew Eldridge^2^, Maria Secrier^2^, Ginny Devonshire^2^, Sriganesh Jammula^2^, Jim Davies^4^, Charles Crichton^4^, Nick Carroll^5^, Richard H.Hardwick^5^, Peter Safranek^5^, Andrew Hindmarsh^5^, Vijayendran Sujendran^5^, Stephen J. Hayes^6,13^, Yeng Ang^6,7,26^, Andrew Sharrocks^26^, Shaun R. Preston^8^, Izhar Bagwan^8^, Vicki Save^9^, Richard J.E. Skipworth^9^, Ted R. Hupp^20^, J. Robert O’Neill^5,9,20^, Olga Tucker^10,29^, Andrew Beggs^10,25^, Philippe Taniere^10^, Sonia Puig^10^, Gianmarco Contino^10^, Timothy J. Underwood^11,12^, Robert C. Walker^11,12^, Ben L. Grace^11^, Jesper Lagergren^14,22^, James Gossage^14,21^, Andrew Davies^14,21^, Fuju Chang^14,21^, Ula Mahadeva^14^, Vicky Goh^21^, Francesca D. Ciccarelli^21^, Grant Sanders^15^, Richard Berrisford^15^, David Chan^15^, Ed Cheong^16^, Bhaskar Kumar^16^, L. Sreedharan^16^ Simon L Parsons^17^, Irshad Soomro^17^, Philip Kaye^17^, John Saunders^6, 17^, Laurence Lovat^18^, Rehan Haidry^18^, Michael Scott^19^, Sharmila Sothi^23^, Suzy Lishman^2, 24^, George B. Hanna^27^, Christopher J. Peters^27^,Krishna Moorthy^27^, Anna Grabowska^28^, Richard Turkington^30^, Damian McManus^30^, Helen Coleman^30^, Russell D Petty^31^ , Freddie Bartlett^32^

^1^ Early Cancer Institute, University of Cambridge, Cambridge, UK

^2^ Cancer Research UK Cambridge Institute, University of Cambridge, Cambridge, UK

^3^ Department of Histopathology, Addenbrooke’s Hospital, Cambridge, UK

^4^Department of Computer Science, University of Oxford, UK, OX1 3QD

^5^Cambridge University Hospitals NHS Foundation Trust, Cambridge, UK, CB2 0QQ

^6^Salford Royal NHS Foundation Trust, Salford, UK, M6 8HD

^7^Wigan and Leigh NHS Foundation Trust, Wigan, Manchester, UK, WN1 2NN

^8^Royal Surrey County Hospital NHS Foundation Trust, Guildford, UK, GU2 7XX

^9^Edinburgh Royal Infirmary, Edinburgh, UK, EH16 4SA

^10^University Hospitals Birmingham NHS Foundation Trust, Birmingham, UK, B15 2GW

^11^University Hospital Southampton NHS Foundation Trust, Southampton, UK, SO16 6YD

^12^Cancer Sciences Division, University of Southampton, Southampton, UK, SO17 1BJ

^13^Faculty of Medical and Human Sciences, University of Manchester, UK, M13 9PL

^14^ Guy’s and St Thomas’s NHS Foundation Trust, London, UK, SE1 7EH

^15^Plymouth Hospitals NHS Trust, Plymouth, UK, PL6 8DH

^16^Norfolk and Norwich University Hospital NHS Foundation Trust, Norwich, UK, NR4 7UY

^17^Nottingham University Hospitals NHS Trust, Nottingham, UK, NG7 2UH

^18^University College London, London, UK, WC1E 6BT

^19^Wythenshawe Hospital, Manchester, UK, M23 9LT

^20^Edinburgh University, Edinburgh, UK, EH8 9YL

^21^King’s College London, London, UK, WC2R 2LS

^22^Karolinska Institute, Stockholm, Sweden, SE-171 77

^23^University Hospitals Coventry and Warwickshire NHS, Trust, Coventry, UK, CV2 2DX

^24^Peterborough Hospitals NHS Trust, Peterborough City Hospital, Peterborough, UK, PE3 9GZ

^25^Institute of Cancer and Genomic sciences, University of Birmingham, B15 2TT

^26^GI science centre, University of Manchester, UK, M13 9PL.

^27^Department of Surgery and Cancer, Imperial College, London, UK, W2 1NY

^28^Queen’s Medical Centre, University of Nottingham, Nottingham, UK

^29^Heart of England NHS Foundation Trust, Birmingham, UK, B9 5SS.

^30^Centre for Cancer Research and Cell Biology, Queen’s University Belfast, Northern Ireland BT7 1NN.

^31^Tayside Cancer Centre, Ninewells Hospital and Medical School, Dundee, DD1 9SY

32 Portsmouth Hospitals NHS Trust, Portsmouth, PO6 3LY

**Gene expression profiling**

H&E sections were annotated for tumour content, with a minimum requirement of 10% viable tumour cell content within the annotated area. Following annotation, 4-5 × 5-μm sections were sectioned and the annotated tumour was macrodissected for RNA isolation. RNA isolation was performed using the Qiagen RNeasy FFPE extraction kit. RNA sequencing was then performed on RNA extracted from the clinical samples using the Illumina Tru-Seq® RNA Exome library preparation kit, followed by sequencing on the Illumina NovaSeq with paired-end reads (75 bp) and 50M total reads per sample. Read alignment was performed using StarAlign to the human reference genome GRCh37/hg19. Gene expression data in Fragments Per Kilobase per Million mapped format (FPKM) was generated using Cufflinks for all genes represented in the human reference genome GRCh37 annotation file. Data quality control was assessed using housekeeping gene coverage (HK metric) whereby only samples with an HK coverage of >/= 2.87 were taken forward for downstream DDIR and clara^T^ analysis.

**Determination of DDIR score**

The DDIR signature was previously developed on cDNA microarray therefore prior to assessment in this study the signature required transfer between the original development platform (microarray) to the current delivery platform (RNASeq). For the signature transfer study, RNA was isolated from 73 FFPE tumour samples followed by matched profiling on the microarray and RNA Exome platforms. Least squares regression was employed to determine any bias between the development platform (microarray) and RNA Exome. The slope and intercept of the regression line were used to establish a new DDIR signature medical decision point (MDP) on the RNASeq platform.   The RNA Exome score that is equivalent to the original microarray score (0.3681) was calculated to be 0.4709. Using this defined threshold of 0.4709 defined DDIR signature status, whereby a score >/= 0.4709 was classified as DDIR positive and <0.4709 classified as DDIR negative (**Supplementary Methods Figure 1**).


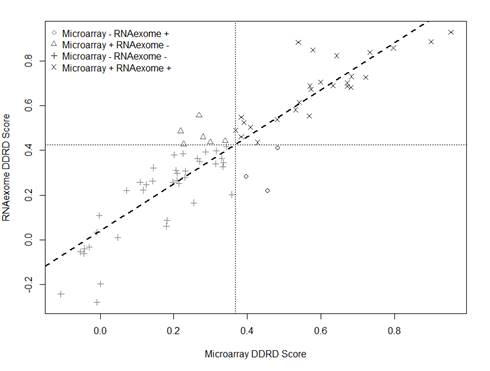


***Supplementary Methods Figure 1.*** *Figure For the assay transfer study, RNA was isolated from 73 FFPE tumour samples followed by matched profiling on both the microarray and RNA Exome platforms. Least squares regression was employed to determine any bias between the development platform (microarray) and RNA Exome. The slope and intercept of the regression line were used to establish a new DDIR Assay medical decision point (MDP) on the RNASeq platform.   The RNA Exome score that is equivalent to the microarray score (0.3681) was calculated to be 0.4709.*

**clara^T^ Total mRNA report**

The raw sequence data in FASTQ format were normalised to FPKM data matrices and processed through Almac Diagnostic Services proprietary analysis pipeline and reporting software for generation of the Version 3.0.0 clara^T^ Total mRNA Report (Almac Diagnostic Services, <https://www.almacgroup.com/diagnostics/claratreport/>). clara^T^ is a software-driven solution, classifying biologically relevant gene expression signatures into a graphical representation. Version 3.0.0 clara^T^ Total mRNA Report, reports on the 10 Hallmarks of Cancer by providing 92 unique gene expression signatures, 100 single targetable genes and >7,000 unique single genes relevant to the ten biologies. Signatures within this report have been reviewed and implemented based on information from the original publications, where possible. Single genes within this report have been selected as analytes relevant to each biology based on the Molecular Signatures Database Hallmark Gene Sets (Liberzon et al. 2015; Copyright ©2004-2017 Broad Institute Inc. and subject to the terms and conditions of the Creative Commons Attribution 4.0 International License). The DDIR signature score for RNAseq data was generated using the clara^T^ software.

***EGFR* Fluorescence in situ hybridisation (FISH)**

*EGFR* FISH was performed and scored using an established protocol[2]. Sections of 4 μm thickness cut from FFPE tumour blocks were mounted on positively charged slides and pre-treated according to the manufacturer’s instructions (Vysis Paraffin Pre-Treatment Reagent Kit II, Abbott Laboratories, Maidenhead, UK).

Briefly, slides were baked at 50^o^C for 2 hours to ensure that the sections were firmly attached to the slides. The slides were then deparaffinised through a xylene and ethanol series using the VP2000 processor.

50 mL of the Heat Pretreatment Solution (reagent 1) from the Cytocell Aquarius® kit was decanted into a plastic Coplin jar with a lid and placed into a water bath set at 98ºC. This was allowed 1 hour to heat. A maximum of 6 slides per preheated Coplin jar of Heat Pretreatment Solution were placed back-to-back and incubated at 98^o^C for 15 minutes in the initial batch of 44 samples tested. This was subsequently changed to 30 minutes. Any samples which required repeat FISH were processed between 30 and 40 minutes. The slides were washed in ambient temperature deionised water for 2 x 3 minutes. The excess water was tapped off and the slides were allowed to semi-air dry.

After ensuring that the Enzyme Reagent from the Cytocell Aquarius® kit was prewarmed to 37^o^C before use, enough drops were added to cover each tissue section. Each slide was flat and the whole section was covered with the reagent. The slides were incubated at room temperature for 30 minutes before draining off the excess reagent and placing the slides in a Coplin jar of deionised water at room temperature for 2 minutes.

The slides were then washed in de-ionised water and were dehydrated in an ethanol series. Once dry, 5-10μl of Vysis *EGFR/CEP7* dual colour probe (Abbott Laboratories, Maidenhead, UK) was applied (dependent on the size of the section) and a glass coverslip was fixed using Scigene Cytobond. The slides were then transferred to a ThermoBrite StatSpin® (Abbott Laboratories) programmed to denature at 85^o^C for 6 minutes, followed by hybridisation at 37^o^C for 16 hours overnight.

Post-hybridisation, slides were washed in 2XSSC/0.3 % Igepal (Sigma-Aldrich Company Ltd Dorset, UK) and air-dried in darkness. Once dried, a nuclear counter stain containing 4’,6-diamidino-2-phenylindole (DAPI) (Vectashield mounting medium, Vector Laboratories, Peterborough, UK) was applied, and a new glass cover slip was attached. Entellan was used to seal the slides for microscope analysis. Slides were stored in darkness at 4^o^C until ready to be microscopically analysed.

FISH scoring analysis was performed by one analyst, using a fluorescent microscope (AXIO Imager M1, Carl Zeiss Microscopy, Cambridge, UK) and images were recorded using CytoVision 7.3.1 software (Leica Biosystems, Newcastle, UK). All areas of the slides were scanned, and three representative areas were selected. These three tumour areas were examined, with 20 cells counted in each area. The figures obtained for *EGFR* and *CEP7* signals were used to calculate both the percentages of cells containing ≤2, 3 or ≥4 copies of *EGFR*, and the ratio between *EGFR* and *CEP7*. In a small number of cases where the result was either borderline or the signal pattern was difficult to interpret, further analysis was carried out by a second independent analyst to clarify the result. *EGFR* status was determined as described in previously published literature[2].

**Immunohistochemistry**

| *Characteristics of the antibodies used for immunohistochemistry* | | | | | | | |
| --- | --- | --- | --- | --- | --- | --- | --- |
| ***Antibody target*** | ***Antibody type*** | ***Antigen retrieval buffer*** | ***Dilution*** | ***Positive control*** | ***Supplier*** | ***Code*** | ***Isotype, clone*** |
| *CD4+ T-cells* | *Mouse monoclonal* | *EDTA* | *1:500* | *Tonsil* | *Abcam* | *Ab133616* | *IgG, EPR6855* |
| *CD8+ T-cells* | *Mouse monoclonal* | *EDTA* | *1:150* | *Tonsil* | *Abcam* | *Ab17147* | *IgG1, 144B* |
| *Foxp3+ T-cells* | *Mouse monoclonal* | *EDTA* | *1:200* | *Tonsil* | *Abcam* | *Ab20034* | *IgG1, 236A/E7* |
| *PD-L1* | *Mouse monoclonal* | *Deionised water* | *1:50* | *Tonsil* | *Dako* | *SK006* | *IgG, 22C3* |
| *Note: Ethylenediaminetetraacetic acid (EDTA) at pH 7.8* | | | | | | | |

**QuPath Analysis**

QuPath was performed using published methodology[3, 4] and *QuPath Version 0.3.2.* Whole slide images (WSI) of immunostained TMA slides for CD4, CD8, FOXP3 and PD-L1 were imported.

For each marker, the following slides were imported:

- 1. Positive and Negative control
  2. Normal
  3. Tumour 1
  4. Tumour 2
  5. Tumour 3
  6. Tumour 4
  7. Tumour 5

For each whole-slide image, NDPI image settings were inputted as below:

*Image provider – .ndpi*

*Set image type – Brightfield (H-DAB)*

*Rotate image – no rotation*

*Auto-generate pyramids selected*

For each WSI, colour devolution was set and optimised initially by using positive and negative control slides. The TMA de-arrayer tool was then used, with optimised settings outlined below.

*TMA core diameter = 1.3mm
Column labels and Row labels = specific based on the slide template
Density threshold = 31%
Bounds scale factor = 105.0*

Each core was manually reviewed by a trained observer to ensure each core was included; boundaries were manually adjusted, and cores were coded as missing if they were unsuitable for analysis i.e. missing core, folded tissue, incorrect tissue type, no scorable tissue (e.g. no tumour tissue).

Simple tissue detection was used to outline the tissue and its boundaries within each digital TMA core. The optimised settings are below:

*Threshold = 226
Requested pixel size: 4µm
Minimum area: 10,000 µm^2^
Maximum fill area: 1,000,000 µm^2^
Dark background (-)
Smooth image (+)
Cleanup with median filter (+)
Expand boundaries (-)
Smooth coordinates (+)
Exclude on boundary (-)
Single annotation (+)*

Tissue detection areas were then manually adjusted to ensure only the relevant tissue was included. For example, loose connective tissue, adipose tissue, lymphoid follicles/aggregates, large blood vessels, and large central gland ‘dead spaces’ were excluded. This was important to ensure an accurate ‘area’ could be calculated for immune cell density measurements.

The method for cell detection/ quantification was determined based on the most appropriate and representative script, as below. For cell detection, the ‘annotation’ created via ‘tissue detection’ (stage 4b) was used, rather than the TMA core (4a). For FOXP3, CD4 and CD8 a single intensity threshold was used (i.e. cells were classed as positive or negative).

*FOXP3 - Positive cell detection*
Positive cell detection was optimised and run as below:

*Detection image: Optical density sum
Requested pixel size: 0.5µm*Nucleus parameters
*Background radius = 8µm
Median filter radius = 0µm
Sigma = 1.5µm
Minimum area = 10µm^2^
Maximum area = 400µm^2^*Intensity Parameters
*Threshold = 0.1
Maximum background intensity = 2
Split by shape (+)
Exclude DAB (membrane staining) (-)*

Cell and General Parameters
*Cell expansion = 5µm
Include cell nucleus (+)
Smooth boundaries (+)
Make measurements (+)*

Intensity Threshold Parameters
*Score compartment: Nucleus: DAB OD mean
Single threshold (+) = 0.25*


*CD8 – Fast cell counts*
Fast cell counts function was optimised and run as below:

Detection Image
*Cell detection channel = Haematoxylin + DAB
Gaussian sigma = 2.5µm
Background radius = 15µm
Use difference of Gaussians (+)*
Thresholding *Cell detection threshold = 0.15
DAB threshold = 0.15*Display
Detection object diameter = 5 pixels

*CD4 – Fast cell counts*
Fast cell counts function was optimised and run as below:

Detection Image
*Cell detection channel = Haematoxylin + DAB
Gaussian sigma = 2µm
Background radius = 15µm
Use difference of Gaussians (-)*
Thresholding *Cell detection threshold = 0.15
DAB threshold = 0.2*Display
Detection object diameter = 5 pixels

*Analysis*

Every core was manually assessed to ensure accuracy. For example, counts were amended to ‘add cells’ that QuPath had missed or ‘subtract cells’ that QuPath had incorrectly counted. QuPath incorrectly counted cells for several reasons such as non-specific background staining, mucinous staining, and stained blood vessels. Thus, a manually adjusted count was calculated for each core.

For analysis of CD4, CD8 and FOXP3, a density was calculated as the number of cells per mm^2^ using corrected counts.

The TMA generally included two cores for each patient. Therefore, the highest score from the two cores should ideally be used for analysis. Where only one core was available that score was used.

For PD-L1 combined positivity score, cores were scored manually by two observers (one an experienced gastrointestinal pathologist) on a shared screen. For patients with two cores an average score was calculated across the cores.

**Statistical analysis**

*Power calculation of study*

The primary research question was: “Do patients with DDIR-positive tumours have longer progression-free survival (PFS) compared with DDIR-negative tumours? Patients will be classified DDIR positive and negative according to methods described. Based on pilot work and other relevant evidence[5, 6], we anticipated observing that ~25% of tumours profiled would be DDR positive.

We planned to identify at least 300 samples that could be analysed by transcriptional profiling with 100 samples from each of the investigated dose levels in the GO2 trial included (Levels A, B and C). These samples would be selected by stratified random sampling to ensure they are representative of the GO2 study populations according to age, sex, performance status and frailty (and its constituent domains).

With 300 samples it was estimated that approximately 225 will be DDIR negative and 75 will be DDR positive. Median follow-up in the GO2 trial population was 12 months with a median PFS (PFS) across all randomised groups was 4.4 months. Using this as a basis for the expected median PFS in the DDR negative group we calculated our study would have good power (>95%) to detect an improved PFS in the DDR positive group of 3 months as shown in **Supplementary Table 1** below. Three months is the minimum clinically relevant difference in PFS that would be considered clinically practice-changing. Changes of this magnitude are not sensitive to small changes in assumptions according to power sensitivity analysis.

**Supplementary Table 2:** Power for various increases in median PFS. These calculations assume the PFS follows the exponential distribution (parameter = 0.1575) with improvements in PFS in the DDR positive group being shown in 1-month increments. The hazard ratios associated with power > 90% are in the range observed in other similar studies [5, 6].

| **N_(DDR–)_** | **N_(DDR+)_** | **Significance level (α)** | **Power**  **(1-β)** | **Median PFS_(DDR–)_**  **(months)** | **Median PFS_(DDR+)_**  **(months)** | **Hazard ratio** |
| --- | --- | --- | --- | --- | --- | --- |
| 225 | 75 | 0.05 | 0.2810 | 4.4 | 5.4 | 0.82 |
| 225 | 75 | 0.05 | 0.7426 | 4.4 | 6.4 | 0.69 |
| 225 | 75 | 0.05 | 0.9578 | 4.4 | 7.4 | 0.60 |
| 225 | 75 | 0.05 | 0.9962 | 4.4 | 8.4 | 0.52 |
| 225 | 75 | 0.05 | 0.9998 | 4.4 | 9.4 | 0.47 |
| 225 | 75 | 0.05 | 0.9999 | 4.4 | 10.4 | 0.42 |

Statistical analyses were conducted according to prespecified statistical analysis plans that were agreed upon before the inspection of any DDIR-stratified outcome data. All clinical-related analyses for ORR, PFS, and OS were performed using R (*version 3.4.1*).

All statistical analyses undertaken for further biological exploration, including Pearson correlation coefficient, Fisher exact test, Student t test, Wilcoxon rank-sum test, Kruskal–Wallis rank-sum test, and one-way ANOVA followed by Tukey honest significance difference test were performed to generate P values for statistical significance using R stats package in R (*version 3.4.0*) and RStudio (*version 1.1383*). In addition to base R packages, ggplot2 R package (version 3.2.1) with other supporting packages, including cowplot (version 0.9.4), ggpubr (version 0.2.3), and grid (version 3.4.0) were used for graphical visualization.

*Data and script availability*

Gene expression dataset and clinicopathologic information in addition to all scripts required to reproduce figures in this article are available from the corresponding authors on request.

**Supplementary Figure S1.** Examples of high and low IHC expression of CD8, CD4, FOXP3 and PD-L1 expression on the Grampian TMA.

**Supplementary Figure S2.** CONSORT diagram of sample selection for RNA sequencing analysis. RNA sequencing was obtained for 32 squamous cell carcinomas and 252 adenocarcinomas. The adenocarcinoma patients were included in subsequent analyses. HER2+; patients who were planned to receive trastuzumab. BSC – best supportive care, ESCC – oesophageal squamous cell carcinoma, GOA – gastroesophageal adenocarcinoma, QC – quality control.

**Supplementary Table 3**. Comparison of baseline characteristics between patients from whom RNA-seq data was available and those in whom it wasn’t. No obvious selection biases were evident based on the GO2 trial stratification factors; sex, dose level, ECOG performance status, planned trastuzumab use, dose reduction. Whole cohort refers to the population who were randomised to receive chemotherapy. *The GO2 trial recorded planned trastuzumab but HER2 status was not formally documented. HER2 testing was performed on the samples with RNA-seq available. These results are available in the main manuscript.

|  | **No RNA-seq available (N=223)** | **RNA-seq available (N=252)** | **Whole cohort (N=475)** | **P-value** |
| --- | --- | --- | --- | --- |
| **Age (Years)** |  |  |  |  |
| Mean (SD) | 75.5 (7.10) | 75.4 (6.47) | 75.5 (6.76) | 0.999 |
| Median (Min, Max) | 77.0 (51.0, 96.0) | 77.0 (52.0, 90.0) | 77.0 (51.0, 96.0) |  |
| **Sex** |  |  |  |  |
| Male | 176 (78.9%) | 187 (74.2%) | 363 (76.4%) | 0.482 |
| Female | 47 (21.1%) | 65 (25.8%) | 112 (23.6%) |  |
| **ECOG PS** |  |  |  |  |
| 0 | 33 (14.8%) | 36 (14.3%) | 69 (14.5%) | 0.981 |
| 1 | 118 (52.9%) | 140 (55.6%) | 258 (54.3%) |  |
| 2+ | 72 (32.3%) | 75 (29.8%) | 147 (30.9%) |  |
| Missing | 0 (0%) | 1 (0.4%) | 1 (0.2%) |  |
| **Dose Level** |  |  |  |  |
| 100% OX | 66 (29.6%) | 85 (33.7%) | 151 (31.8%) | NA |
| 80% OX | 78 (35.0%) | 75 (29.8%) | 153 (32.2%) |  |
| 60% OX | 79 (35.4%) | 92 (36.5%) | 171 (36.0%) |  |
| **Primary site** |  |  |  |  |
| Oesophagus | 70 (31.4%) | 86 (34.1%) | 156 (32.8%) | 0.364 |
| GOJ | 49 (22.0%) | 72 (28.6%) | 121 (25.5%) |  |
| Gastric | 102 (45.7%) | 94 (37.3%) | 196 (41.3%) |  |
| Missing | 2 (0.9%) | 0 (0%) | 2 (0.4%) |  |
| **Planned Trastuzumab*** |  |  |  |  |
| Positive | 12 (5.4%) | 15 (6.0%) | 27 (5.7%) | 0.965 |
| Negative/Unknown | 211 (94.6%) | 237 (94.0%) | 448 (94.3%) |  |
| **Metastases present** |  |  |  |  |
| Yes | 149 (66.8%) | 170 (67.5%) | 319 (67.2%) | 0.989 |
| No | 74 (33.2%) | 82 (32.5%) | 156 (32.8%) |  |
| **GO2 Frailty Group** |  |  |  |  |
| Not frail | 38 (17.0%) | 48 (19.0%) | 86 (18.1%) | 0.852 |
| Slightly frail | 48 (21.5%) | 62 (24.6%) | 110 (23.2%) |  |
| Severely frail | 137 (61.4%) | 141 (56.0%) | 278 (58.5%) |  |
| Missing | 0 (0%) | 1 (0.4%) | 1 (0.2%) |  |
| **GO2 Frailty Score** |  |  |  |  |
| Mean (SD) | 2.96 (1.47) | 2.77 (1.36) | 2.86 (1.42) | 0.341 |
| Median (Min, Max) | 3.00 (0, 7.00) | 3.00 (0, 8.00) | 3.00 (0, 8.00) |  |
| Missing | 0 (0%) | 1 (0.4%) | 1 (0.2%) |  |
| **Arm of study** |  |  |  |  |
| Uncertain | 8 (3.6%) | 11 (4.4%) | 19 (4.0%) | 0.911 |
| Certain | 215 (96.4%) | 241 (95.6%) | 456 (96.0%) |  |

**Supplementary Figure S3** – Overall survival in the GO2 adenocarcinoma population according to whether RNA sequencing data was available or not. Median OS was 7.0 months (95% CI; 6.1-8.0) in the population without RNA sequencing data vs 7.6 (95% CI; 7.1-8.6) in the population with, HR 0.94 (95% CI; 0.76-1.17), p=0.6.


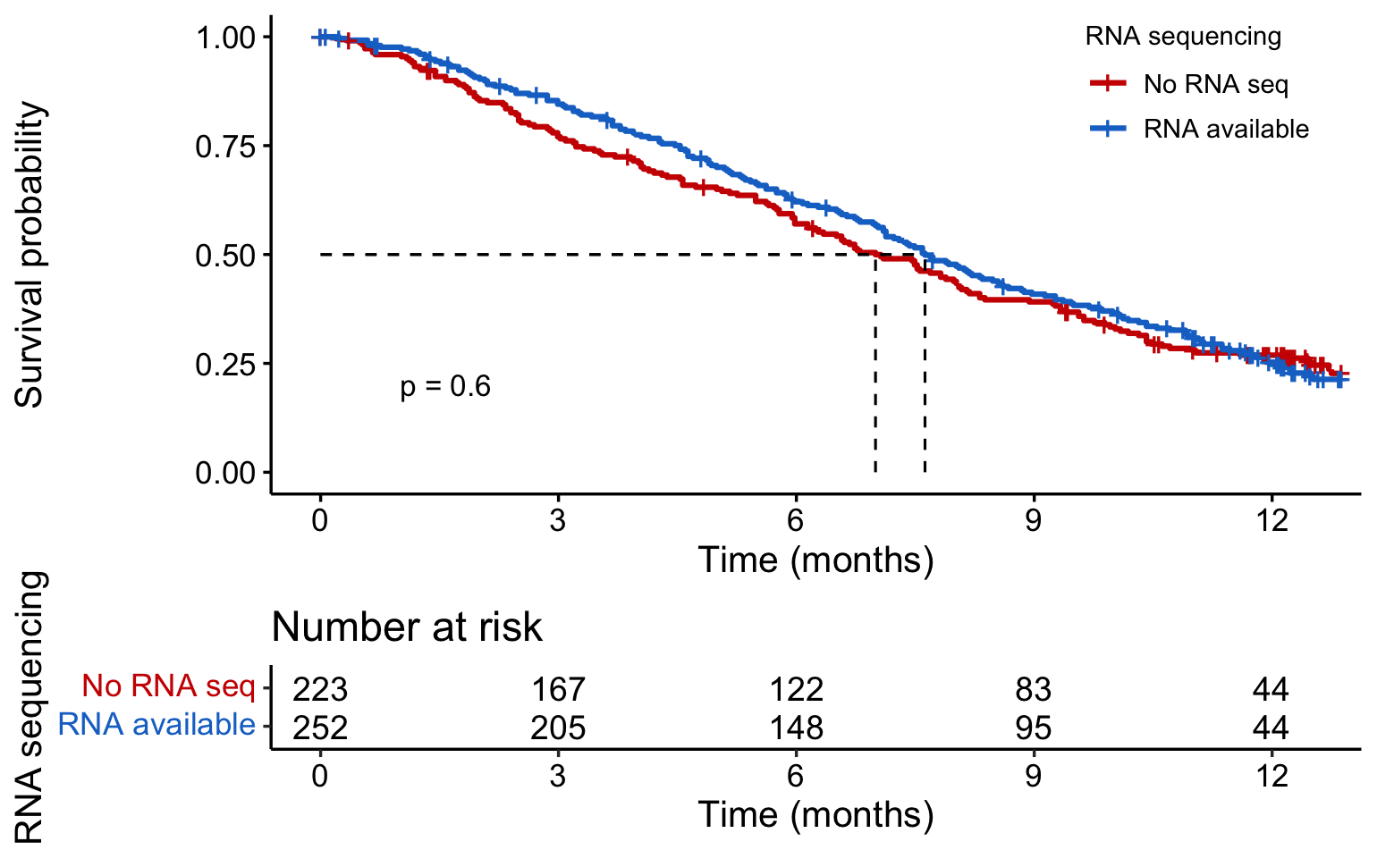


**Supplementary Figure S4.** CONSORT diagram of patient selection for assessment of response according to DDIR status.

**Supplementary Figure S5:** Radiological response (partial or complete response) according to DDIR status in the GOA population who received chemotherapy in the GO2 trial.

**Supplementary Table 4.** Radiological response rate (complete and partial responses) in the GO2 adenocarcinoma cohort according to dose level for DDIR positive and DDIR negative samples.

|  | **Negative** | | | **Positive** | | |  |
| --- | --- | --- | --- | --- | --- | --- | --- |
|  | **100% OX (N=60)** | **80% OX (N=55)** | **60% OX (N=64)** | **100% OX (N=10)** | **80% OX (N=7)** | **60% OX (N=12)** | **P-value** |
| **Response** |  |  |  |  |  |  |  |
| No response | 38 (63.3%) | 40 (72.7%) | 49 (76.6%) | 4 (40.0%) | 4 (57.1%) | 6 (50.0%) | 0.107 |
| Response | 22 (36.7%) | 15 (27.3%) | 15 (23.4%) | 6 (60.0%) | 3 (42.9%) | 6 (50.0%) |  |

**Supplementary Table 5.** Radiological response rate (complete and partial responses) in the GO2 adenocarcinoma cohort according to DDIR status within each dose level.

|  | **100% OX** | | **80% OX** | | **60% OX** | |  |
| --- | --- | --- | --- | --- | --- | --- | --- |
|  | **Negative (N=60)** | **Positive (N=10)** | **Negative (N=55)** | **Positive (N=7)** | **Negative (N=64)** | **Positive (N=12)** | **P-value** |
| **Response** |  |  |  |  |  |  |  |
| No response | 38 (63.3%) | 4 (40.0%) | 40 (72.7%) | 4 (57.1%) | 49 (76.6%) | 6 (50.0%) | 0.107 |
| Response | 22 (36.7%) | 6 (60.0%) | 15 (27.3%) | 3 (42.9%) | 15 (23.4%) | 6 (50.0%) |  |

**Supplementary Figure S6.** CONSORT diagram of patient selection for assessment of survival according to DDIR status.

**Supplementary Figure S7.** Cox-regression analysis for progression-free survival in the GO2 adenocarcinoma population.

**
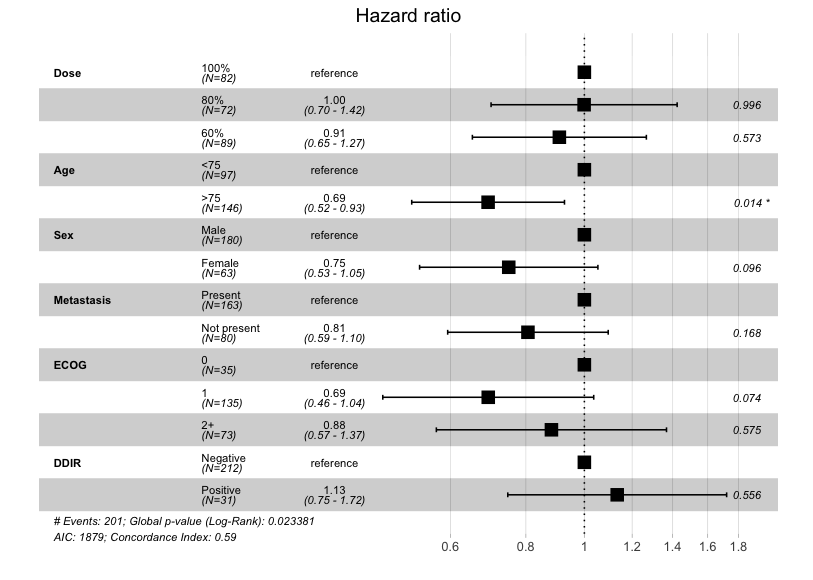
**

**Supplementary Figure S8 (A)** Cox regression analysis of overall survival in the GO2 adenocarcinoma population according to stratification factors and DDIR status. (**B)** Cox regression analysis of overall survival in the DDIR-positive GO2 adenocarcinoma population according to stratification factors.

**Supplementary Figure S9.** Progression-free survival (PFS) in the DDIR-positive population in the GO2 trial according to dose level. *n=22 as one patient did not have a PFS documented.

**
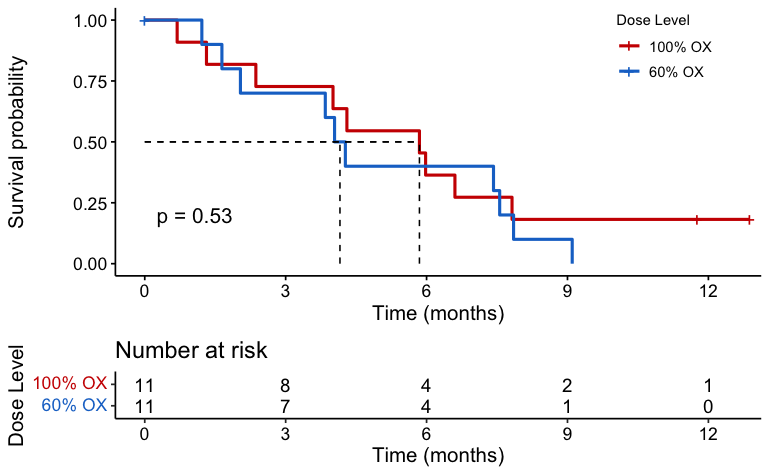
**

**Supplementary Figure S10.** Overall survival in the DDIR-positive population in the GO2 trial according to dose level.

**
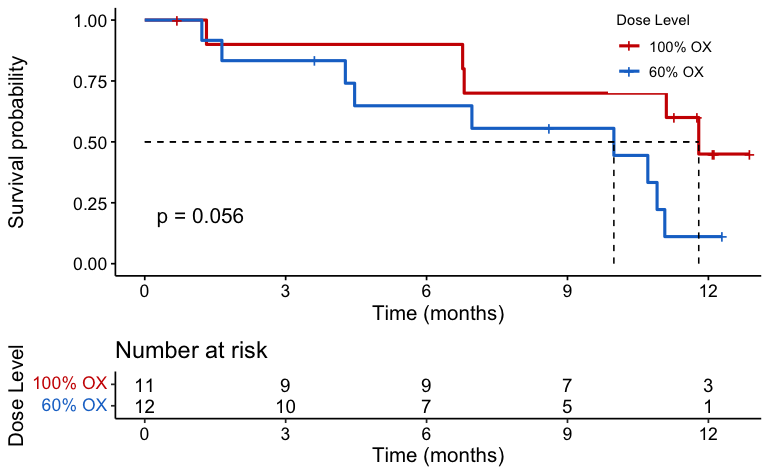
**

**Supplementary Figure S11.** Quality of life (visual analogue scale) for DDIR-positive patients in dose levels A (100% OX) and C (60% OX) during 1 year of follow-up.


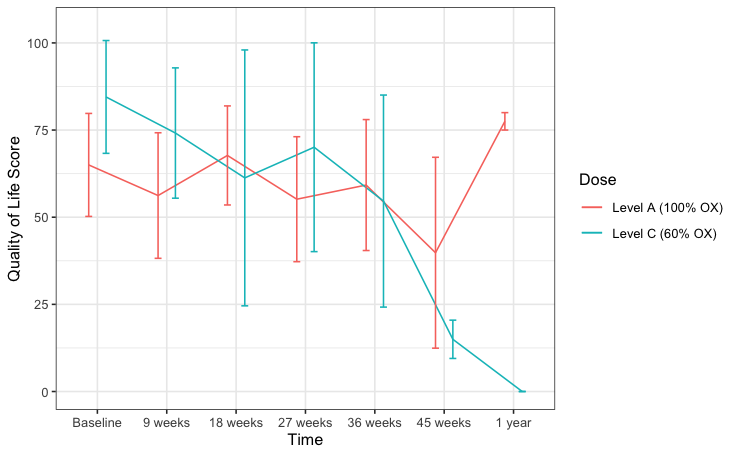


**Supplementary Table 6.** Overall treatment utility (OTU) for DDIR-positive patients in dose levels A (100% OX) and C (60% OX).

| **Overall Treatment Utility** | **100% OX (n=11)** | **60% OX (n=12)** | **p-value** |
| --- | --- | --- | --- |
| **Good** | 4 (36.4%) | 4 (33.3%) | **0.418** |
| **Intermediate** | 2 (18.2%) | 5 (41.7%) |  |
| **Poor** | 5 (45.5%) | 3 (25.0%) |  |

**Supplementary Figure S12.** Cox-regression analysis for overall survival in the DDIR-negative population.


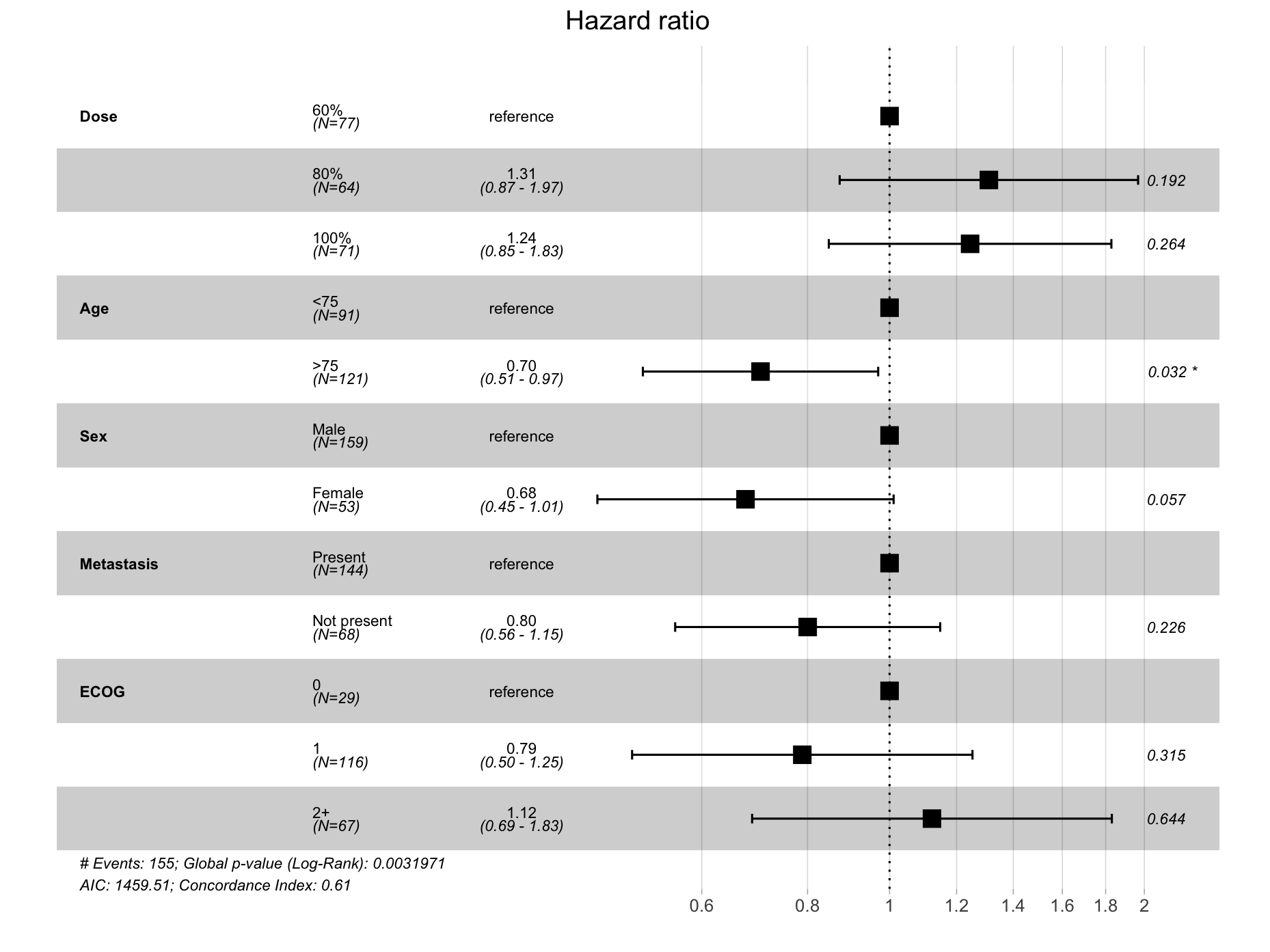


**Supplementary Figure S13.** Quality of life (VAS) in the DDIR-negative population according to dose level.

**
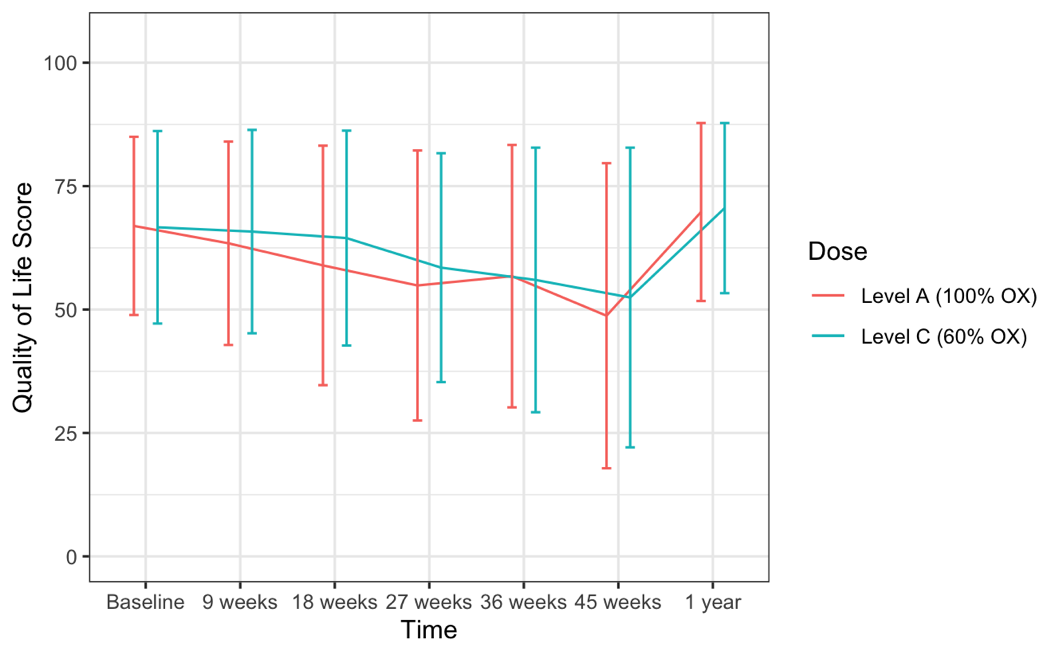
**

**Supplementary Figure S14.** Overall Treatment Utility (OTU) in the DDIR-negative population according to dose level.

**
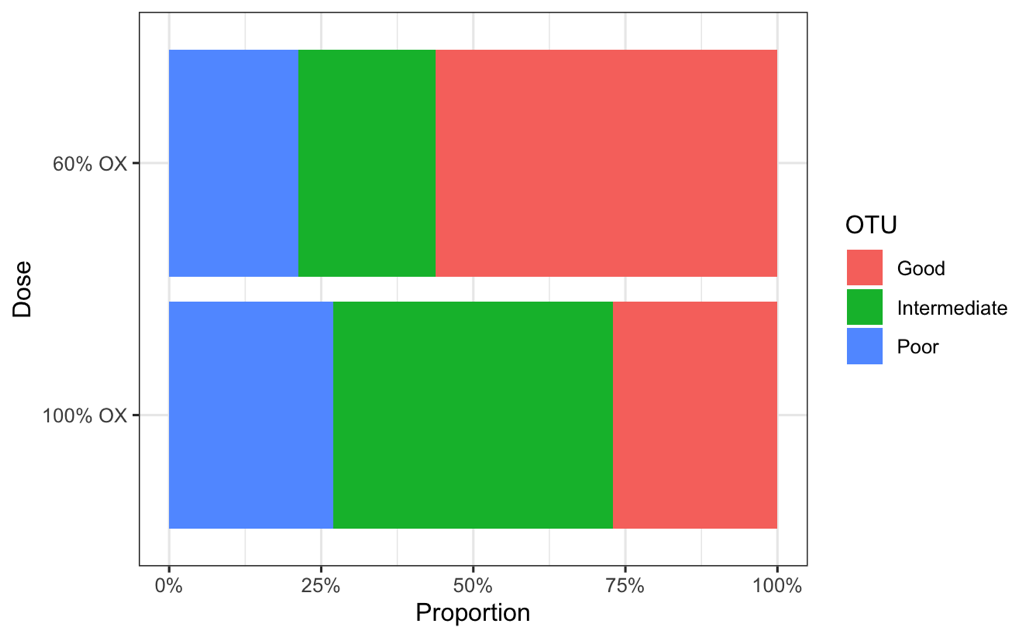
**

**Supplementary Figure S15.** MSI Signature Score compared to mismatch repair (MMR) deficiency immunohistochemistry (n=190) in the GO2 adenocarcinoma population. Thirteen (6.8%) had MMR deficiency on immunohistochemistry (IHC). 29 additional samples had no tumour visible and 5 failed IHC testing.

**
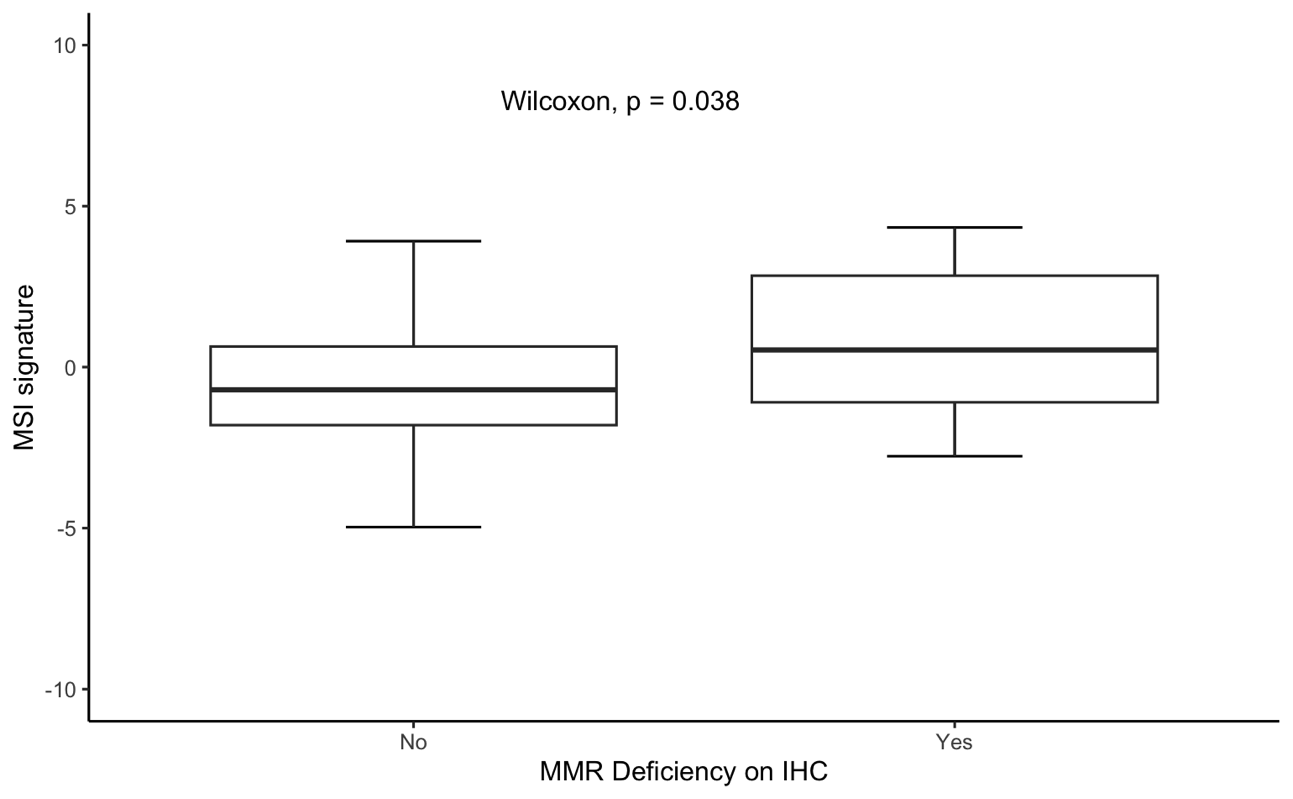
**

**Supplementary Table 7.** Baseline characteristics of the GO2 population who received 100% OX (dose level A) according to DDIR status. PS – performance status, SD – standard deviation.

|  | **DDIR Negative (n=71)** | **DDIR Positive (n=11)** | **p-value** |
| --- | --- | --- | --- |
| **Age (years)** |  |  |  |
| Mean (SD) | 74.5 (6.58) | 78.7 (5.16) | **<0.001** |
| Median (Min, Max) | 75.0 (57.0, 86.0) | 80.0 (65.0, 83.0) |  |
| **Sex** |  |  |  |
| Male | 58 (81.7%) | 8 (72.7%) | 0.772 |
| Female | 13 (18.3%) | 3 (27.3%) |  |
| **ECOG PS** |  |  |  |
| 0 | 11 (15.5%) | 2 (18.2%) | NA |
| 1 | 39 (54.9%) | 6 (54.5%) |  |
| 2+ | 21 (29.6%) | 3 (27.3%) |  |
| **Primary site** |  |  |  |
| Oesophagus | 16 (22.5%) | 2 (18.2%) | 0.058 |
| GOJ | 29 (40.8%) | 1 (9.1%) |  |
| Gastric | 26 (36.6%) | 8 (72.7%) |  |
| **Planned trastuzumab** |  |  |  |
| Yes | 3 (4.2%) | 1 (9.1%) | 1 |
| No/Unknown | 68 (95.8%) | 10 (90.9%) |  |
| **Metastasis** |  |  |  |
| Yes | 53 (74.6%) | 7 (63.6%) | 0.688 |
| No | 18 (25.4%) | 4 (36.4%) |  |
| **GO2 Frailty Group** |  |  |  |
| Not frail | 13 (18.3%) | 0 (0%) | 0.217 |
| Slightly frail | 17 (23.9%) | 2 (18.2%) |  |
| Severely frail | 41 (57.7%) | 9 (81.8%) |  |
| **GO2 Frailty Score** |  |  |  |
| Mean (SD) | 2.75 (1.25) | 3.45 (1.04) | **0.008** |
| Median (Min, Max) | 3.00 (0, 5.00) | 3.00 (2.00, 5.00) |  |

**Supplementary Table 8**. The hierarchical clusters according to Clara^T^ signature biologies based on the RNA sequencing data obtained from the GO2 GOA population. EMT - epithelial mesenchymal transition), HRD – homologous recombination deficiency, TGF-β - transforming growth factor β.

| **Cluster** | **Characteristics** |
| --- | --- |
| **1 (n=33)** | TGF-β + EMT + Angiogenesis |
| **2 (n=38)** | HRD, cell cycle checkpoints with IFNγ expression |
| **3 (n=55)** | EMT + inflammation |
| **4 (n=41)** | Proliferation |
| **5 (n=51)** | HRD, cell cycle checkpoints without IFNγ expression |
| **6 (n=34)** | No specific biologies |

**Supplementary Figure S16.** Hierarchical clustering heat map of Almac Clara^T^ signatures derived from the RNA sequencing data from the GO2 tumour samples. Patients are represented on the top column and signatures grouped according to relevant Hallmark of Cancer are represented by rows. The data includes 252 patients with GOA and 32 patients with squamous cell carcinoma. Clusters 1-3 are inflammatory and clusters 4-6 non-inflammatory. The boxes highlight the key upregulated signatures in each cluster.

**Supplementary Figure S17.** Raw Clara^T^ DDIR signature score according to each RNA-sequencing hierarchical cluster in the GO2 GOA population (n=252).


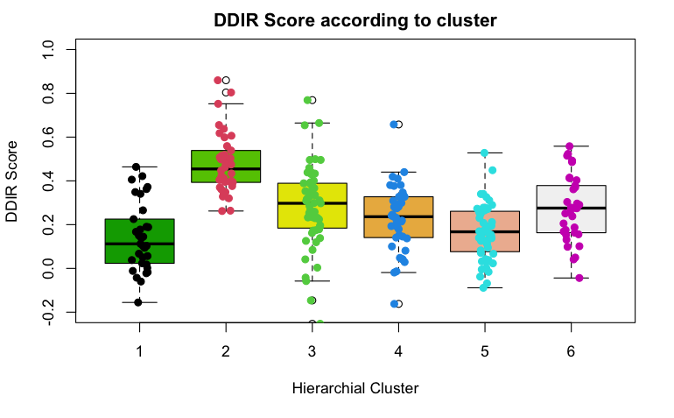


Hierarchical Cluster

**Supplementary Table 9.** DDIR status distribution according to RNA-sequencing hierarchical cluster in the GO2 GOA population (n=252).

|  | **DDIR Call** | |  |
| --- | --- | --- | --- |
|  | **Positive** | **Negative** | **% DDIR Positive** |
| **Cluster 1** | 0 | 33 | 0% |
| **Cluster 2** | 18 | 20 | 47.4% |
| **Cluster 3** | 6 | 49 | 10.9% |
| **Cluster 4** | 1 | 40 | 2.4% |
| **Cluster 5** | 1 | 50 | 2.0% |
| **Cluster 6** | 5 | 29 | 14.7% |

**Supplementary Table 10.** Signatures which predicted response and a dose-dependent survival at a significance level of p<0.05 in the GO2 population. The signatures initially included were DDIR[7], NFκB [8], T cell inflamed GEP[9], TGCA CSF1 response[10] and CTLA4 Response Signature[11]. Genes from each signature were included in a gene comparison analysis to identify shared genes between signatures (<https://bioinformatics.psb.ugent.be/> webtools/Venn).

| **DDIR Signature[7]** | **NFκB signature [8]** | **T cell inflamed GEP[9]** | **TGCA CSF1 response[10]** | **CTLA4 Response Signature[11]** |
| --- | --- | --- | --- | --- |
| \| *CXCL10* \| \| --- \| \| *MX1* \| \| *IDO1* \| \| *CD2* \| \| *GBP5* \| \| *PRAME* \| \| *ITGAL* \| \| *LRP4* \| \| *APOL3* \| \| *CDR1* \| \| *FYB* \| \| *TSPAN7* \| \| *RAC2* \| \| *KLHDC7B* \| \| *GRB14* \| \| *KIF26A* \| \| *CD274* \| \| *CD109* \| \| *ETV7* \| \| *MFAP5* \| \| *OLFM4* \| \| *PI15* \| \| *FOSB* \| \| *FAM19A5* \| \| *NLRC5* \| \| *PRICKLE1* \| \| *EGR1* \| \| *CLDN10* \| \| *ADAMTS4* \| \| *SP140L* \| \| *ANXA1* \| \| *RSAD2* \| \| *ESR1* \| \| *IKZF3* \| \| *EGFR* \| \| *NAT1* \| \| *LATS2* \| \| *CYP2B6* \| \| *PTPRC* \| \| *PPP1R1A* \| | \| *GBP1* \| \| --- \| \| *PSMB9* \| \| *IRF1* \| \| *TAP1* \| \| *TNFAIP3* \| \| *CCL5* \| \| *PSMB8* \| \| *IL32* \| \| *SH2B3* \| \| *NFKBIE* \| | \| *IFNγ* \| \| --- \| \| *STAT1* \| \| *CCR5* \| \| *CXCL9* \| \| *CXCL10* \| \| *CXCL11* \| \| *IDO1* \| \| *PRF1* \| \| *GZMA* \| \| *MHCII HLA-DRA* \| | \| *CTSL1* \| \| --- \| \| *CD163* \| \| *FCGR3a* \| \| *FCGR2a* \| \| *CSF1R* \| | \| *IDO1* \| \| --- \| \| *TNNC2* \| \| *ITM2A* \| \| *CXCL10* \| \| *HPGD* \| \| *HLA-DQA1* \| \| *GBP1* \| \| *CKMT2* \| \| *FGL2* \| \| *GZMB* \| \| *PRF1* \| \| *OR7A5* \| \| *CXCL11* \| \| *FAIM3* \| \| *CXCL9* \| \| *CD8A* \| \| *CCL5* \| \| *NKG7* \| \| *OMD* \| \| *IGLA* \| \| *CD38* \| \| *TNXB* \| \| *APOL3* \| \| *CCL4* \| |

**Supplementary Table 11.** Overlap of genes involved in the signatures associated with improved response rate and dose-dependent survival in the GO2 GOA population.

| **Signatures** | **Number Shared** | **Genes** |
| --- | --- | --- |
| CTLA4 response/DDIR/TcellGEP | 2 | *CXCL10*  *IDO1* |
| CTLA4 response/DDIR | 1 | *APOL3* |
| CTLA4 response/NFκB | 2 | *GBP1*  *CCL5* |
| CTLA4 response/TcellGEP | 3 | *PRF1*  *CXCL11*  *CXCL9* |

**Supplementary Table 12.** Gepia 2.0 T-cell signatures correlated against *CXCL10* RNA expression, *CXCL10/IDO1, CXCL10/CCL5* or DDIR signature in oesophageal and gastric cancer tumours.

|  |  | **Pearson correlation vs T-cell signature** | | | |
| --- | --- | --- | --- | --- | --- |
| **TCGA T-cell Signature** | **Genes** | ***CXCL10*** | ***CXCL10/IDO1*** | ***CXCL10/CCL5*** | **DDIR Signature** |
| Naïve T-cell | *CCR7*  *LEF1*  *TCF7*  *SELL* | 0.07 (p=0.091) | 0.29 (p<0.001) | 0.37 (p<0.001) | 0.57 (p<0.001) |
| Effector T-cell | *CX3CR1*  *FGFBP2*  *FCGR3A* | 0.24 (p<0.001) | 0.42 (p<0.001) | 0.51 (p<0.001) | 0.56 (p<0.001) |
| Effector memory T-cell | *PDCD1*  *DUSP4*  *GZMK*  *GZMA*  *IFNG* | 0.38 (p<0.001) | 0.71 (p<0.001) | 0.77 (p<0.001) | 0.58 (p<0.001) |
| Central memory T-cell | *CCR7*  *SELL*  *IL7R* | 0.18 (p<0.001)) | 0.41 (p<0.001) | 0.49 (p<0.001) | 0.68 (p<0.001) |
| Resident memory T-cell | *CD69*  *ITGAE*  *CXCR6*  *MYADM* | 0.29 (p<0.001) | 0.56 (p<0.001) | 0.65 (p<0.001) | 0.73 (p<0.001) |
| Exhausted T-cell | *HAVCR2*  *TIGIT*  *LAG3*  *PDCD1*  *CXCL13*  *LAYN* | 0.37 (p<0.001) | 0.71 (p<0.001) | 0.81 (p<0.001) | 0.78 (p<0.001) |
| Resting Treg T-cell | *FOXP3*  *IL2RA* | 0.37 (p<0.001) | 0.68 (p<0.001) | 0.72 (p<0.001) | 0.74 (p<0.001) |
| Effector Treg T-cell | *FOXP3*  *CTLA4*  *CCR8*  *TNFRSF9* | 0.38 (p<0.001) | 0.69 (p<0.001) | 0.73 (p<0.001) | 0.74 (p<0.001) |
| Th1-like | *CXCL13*  *HAVCR2*  *IFNG*  *CXCR3*  *BHLHE40*  *CD4* | 0.38 (p<0.001) | 0.73 (p<0.001) | 0.81 (p<0.001) | 0.78 (p<0.001) |
| All signatures | All above genes | 0.35 (p<0.001) | 0.68 (p<0.001) | 0.77 (p<0.001) | 0.8 (p<0.001) |

**Supplementary Figure S18.** MCP immune cell tumour microenvironment populations according to CXCL10/CCL5 RNA signature expression. High – top 25% of expressors.

**Supplementary Table 13.** Radiological response according to *CXCL10/CCL5* signature expression in the GO2 adenocarcinoma population.

|  | **Rest of population (N=148)** | **Top quartile (N=51)** | **P-value** |
| --- | --- | --- | --- |
| **Response** |  |  |  |
| No response | 106 (71.6%) | 28 (54.9%) | 0.043 |
| Response | 42 (28.4%) | 23 (45.1%) |  |

**Supplementary Figure S19.** Cox regression analysis for overall survival incorporating the combined *CXCL10/CCL5* RNA expression signature in the GO2 GOA population treated with Dose Level A (100% OX).


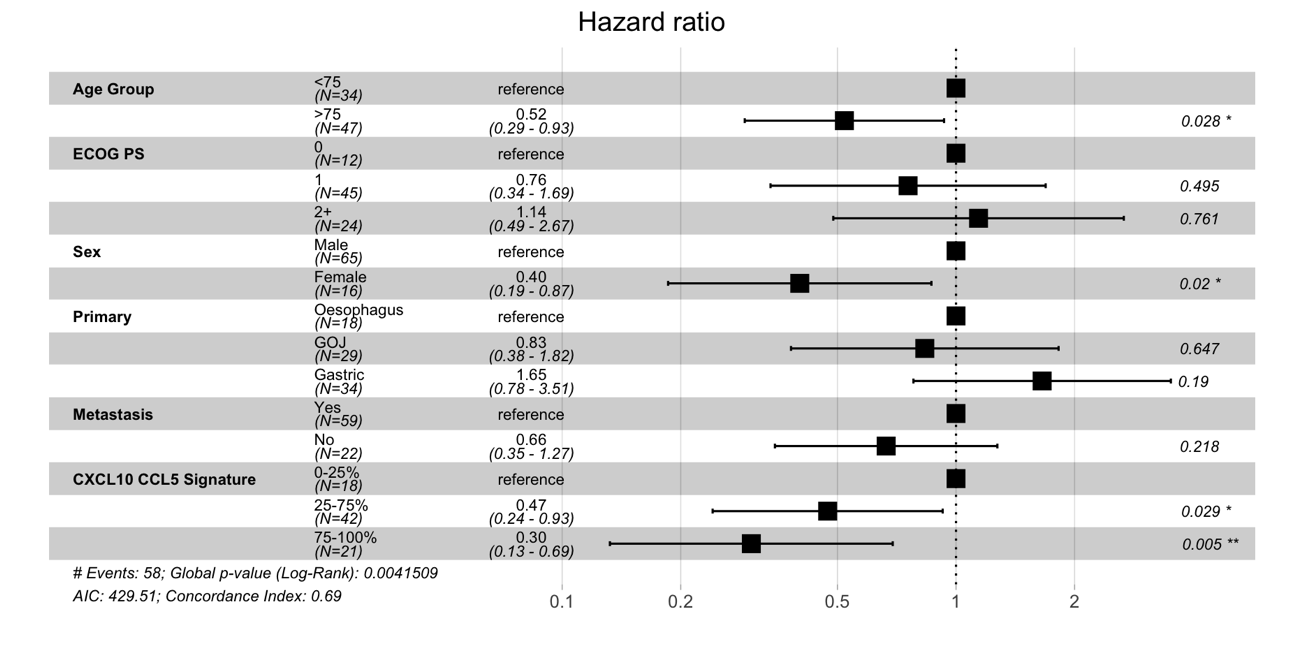


**Supplementary Table 14.** OCCAMS validation cohort demographics.

|  | **Overall (N=306)** |
| --- | --- |
| **Age At Diagnosis** |  |
| Mean (SD) | 67.0 (9.44) |
| Median (Min, Max) | 67.6 (40.9, 87.7) |
| **Patient Gender** |  |
| Female | 44 (14.4%) |
| Male | 261 (85.3%) |
| Unknown | 1 (0.3%) |
| **ECOG Performance Status** |  |
| 0 | 180 (58.8%) |
| 1 | 84 (27.5%) |
| 2 | 10 (3.3%) |
| Unknown | 25 (8.2%) |
| **Biopsy Histology** |  |
| Adenocarcinoma | 299 (97.7%) |
| High-grade dysplasia | 1 (0.3%) |
| High-grade dysplasia with intramucosal carcinoma | 6 (2.0%) |
| **Pre-treatment T Stage** |  |
| T1 | 25 (8.2%) |
| T2 | 52 (17.0%) |
| T3 | 201 (65.7%) |
| T4 | 12 (3.9%) |
| Tx | 6 (2.0%) |
| No evidence of tumour/Cannot be assessed | 8 (2.6%) |
| Not recorded | 3 (1.0%) |
| **Pre-treatment N Stage** |  |
| N0 | 107 (35.0%) |
| N1 | 119 (38.9%) |
| N2 | 59 (19.3%) |
| N3 | 12 (3.9%) |
| Nx | 8 (2.6%) |
| **Pre-treatment M Stage** |  |
| M0 | 256 (83.7%) |
| M1 | 25 (8.2%) |
| Mx | 23 (7.5%) |
|  |  |

**Supplementary Figure S20.** OCCAMS RNA-sequencing *CXCL10* expression (FPKM) vs MCP Cytotoxic T-lymphocyte score.

**
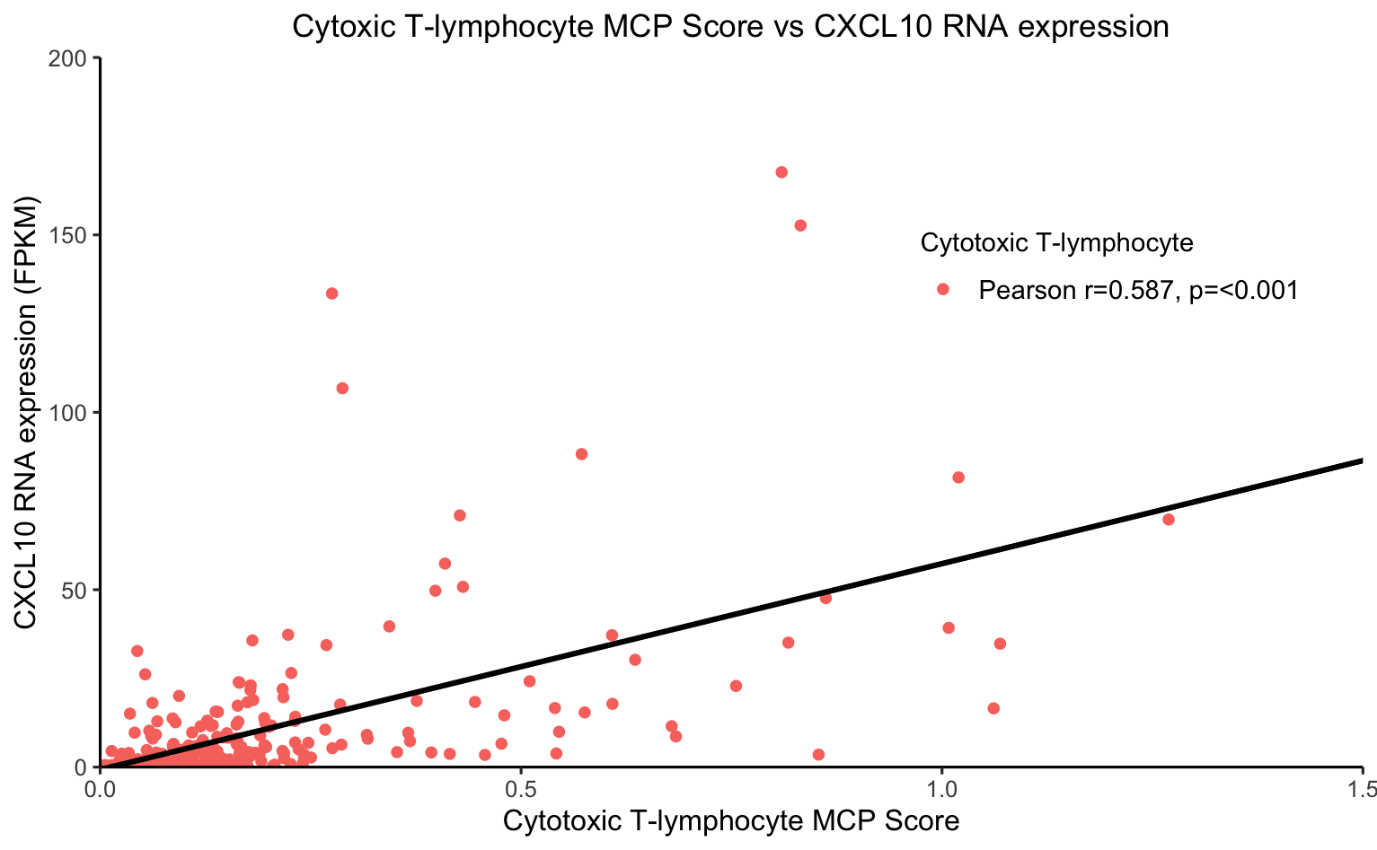
**

**Supplementary Figure S21.** Consensus Pathway analysis with high confidence of the genes included in the DDIR signature. A z-threshold of 30 for intermediate nodes was used. Black writing – seed nodes, pink writing – intermediate nodes.

**
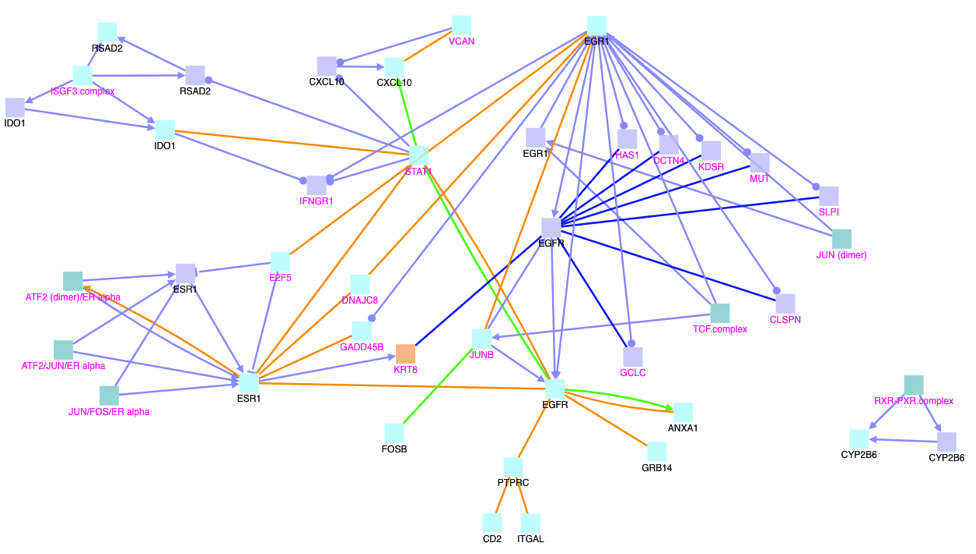
**

**
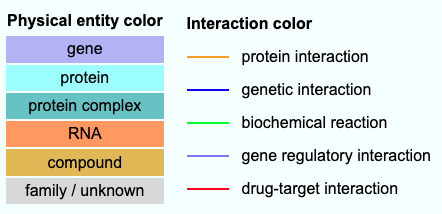
**

**Supplementary Figure S22.** Overall survival according to *EGFR* RNA expression (FPKM) in the GO2 GOA population. Median OS in low, medium and high *EGFR* expressors was 9.3 months (95% CI; 6.3-11.1) vs 7.9 months (95% CI; 7.9-7.1) vs 7.6 months (95% CI; 7.6-6.6).


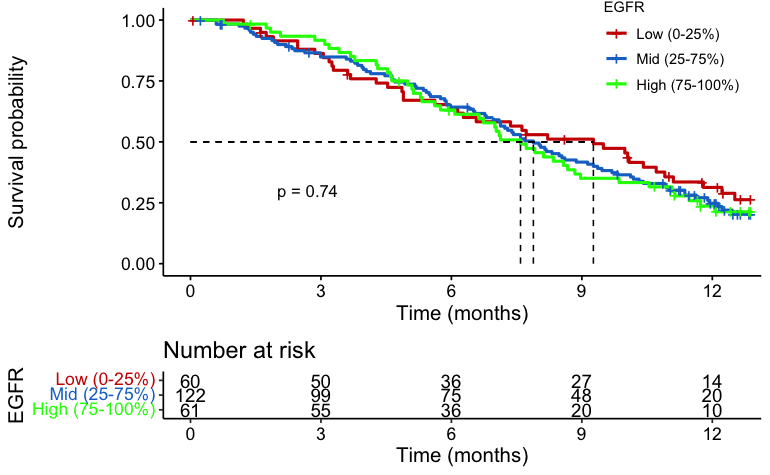


**Supplementary Figure S23.** Correlation between *EGFR* RNA expression and DDIR Score in the GO2 GEA population. The red line denotes DDIR positivity. The blue line denotes the level of *EGFR* RNA expression which excludes DDIR positivity. Correlation – R = -0.251 (95% CI; -0.364—0.132), p<0.0001.

**Supplementary Table 15.** Baseline demographics in the GO2 GOA population according to whether or not FISH analysis was performed. Whole population is adenocarcinoma population in the ‘likely to benefit’ arm.

|  | **FISH not performed (N=332)** | **FISH performed (N=143)** | **GO2 Population (N=475)** | **P-value** |
| --- | --- | --- | --- | --- |
| **Age (Years)** |  |  |  |  |
| Mean (SD) | 75.0 (6.87) | 76.4 (6.44) | 75.5 (6.76) | 0.142 |
| Median (Min, Max) | 76.0 (51.0, 96.0) | 77.0 (52.0, 90.0) | 77.0 (51.0, 96.0) |  |
| **Sex** |  |  |  |  |
| Male | 251 (75.6%) | 112 (78.3%) | 363 (76.4%) | 0.815 |
| Female | 81 (24.4%) | 31 (21.7%) | 112 (23.6%) |  |
| **ECOG PS** |  |  |  |  |
| 0 | 51 (15.4%) | 18 (12.6%) | 69 (14.5%) | 0.278 |
| 1 | 169 (50.9%) | 89 (62.2%) | 258 (54.3%) |  |
| 2+ | 111 (33.4%) | 36 (25.2%) | 147 (30.9%) |  |
| Missing | 1 (0.3%) | 0 (0%) | 1 (0.2%) |  |
| **Dose Level** |  |  |  |  |
| 100% OX | 105 (31.6%) | 46 (32.2%) | 151 (31.8%) | 0.995 |
| 80% OX | 109 (32.8%) | 44 (30.8%) | 153 (32.2%) |  |
| 60% OX | 118 (35.5%) | 53 (37.1%) | 171 (36.0%) |  |
| **Primary site** |  |  |  |  |
| Oesophagus | 111 (33.4%) | 45 (31.5%) | 156 (32.8%) | 0.985 |
| GOJ | 82 (24.7%) | 39 (27.3%) | 121 (25.5%) |  |
| Gastric | 137 (41.3%) | 59 (41.3%) | 196 (41.3%) |  |
| Missing | 2 (0.6%) | 0 (0%) | 2 (0.4%) |  |
| **HER2 status** |  |  |  |  |
| Positive | 22 (6.6%) | 5 (3.5%) | 27 (5.7%) | 0.401 |
| Negative/Unknown | 310 (93.4%) | 138 (96.5%) | 448 (94.3%) |  |
| **Metastases present** |  |  |  |  |
| Yes | 225 (67.8%) | 94 (65.7%) | 319 (67.2%) | 0.91 |
| No | 107 (32.2%) | 49 (34.3%) | 156 (32.8%) |  |
| **GO2 Frailty Group** |  |  |  |  |
| Not frail | 65 (19.6%) | 21 (14.7%) | 86 (18.1%) | 0.389 |
| Slightly frail | 69 (20.8%) | 41 (28.7%) | 110 (23.2%) |  |
| Severely frail | 197 (59.3%) | 81 (56.6%) | 278 (58.5%) |  |
| Missing | 1 (0.3%) | 0 (0%) | 1 (0.2%) |  |
| **GO2 Frailty Score** |  |  |  |  |
| Mean (SD) | 2.86 (1.45) | 2.86 (1.35) | 2.86 (1.42) | 1 |
| Median (Min, Max) | 3.00 (0, 7.00) | 3.00 (0, 8.00) | 3.00 (0, 8.00) |  |
| Missing | 1 (0.3%) | 0 (0%) | 1 (0.2%) |  |
| **Likely to benefit** |  |  |  |  |
| Uncertain | 14 (4.2%) | 5 (3.5%) | 19 (4.0%) | 0.935 |
| Certain | 318 (95.8%) | 138 (96.5%) | 456 (96.0%) |  |

**Supplementary Figure S24.** CONSORT diagram for tissue selection for EGFR FISH in the GO2 trial GOA tumour samples.

Stratified list of patients according to DDIR status provided by Leeds CTU statisticians (1:4 ratio – total 153: 31 DDIR positive, 122 DDIR negative)

**Supplementary Table 16.** Demographics according to successful EGFR FISH status in the GO2 GOA population.

|  | **FISH Positive** | | **FISH Negative** |  |
| --- | --- | --- | --- | --- |
|  | **FISH Amplified (N=11)** | **FISH HP (N=19)** | **FISH Negative (N=94)** | **P-value** |
| **Age (years)** |  |  |  |  |
| Mean (SD) | 76.3 (4.71) | 72.7 (8.14) | 76.8 (6.03) | **0.04** |
| Median (Min, Max) | 77.0 (69.0, 83.0) | 75.0 (52.0, 83.0) | 77.0 (57.0, 90.0) |  |
| **Sex** |  |  |  |  |
| Male | 9 (81.8%) | 15 (78.9%) | 72 (76.6%) | 0.912 |
| Female | 2 (18.2%) | 4 (21.1%) | 22 (23.4%) |  |
| **ECOG PS** |  |  |  |  |
| 0 | 1 (9.1%) | 1 (5.3%) | 12 (12.8%) | 0.81 |
| 1 | 8 (72.7%) | 12 (63.2%) | 56 (59.6%) |  |
| 2+ | 2 (18.2%) | 6 (31.6%) | 26 (27.7%) |  |
| **Dose Level** |  |  |  |  |
| 100% OX | 4 (36.4%) | 6 (31.6%) | 31 (33.0%) | 0.459 |
| 80% OX | 5 (45.5%) | 8 (42.1%) | 26 (27.7%) |  |
| 60% OX | 2 (18.2%) | 5 (26.3%) | 37 (39.4%) |  |
| **Site of primary** |  |  |  |  |
| Oesophagus | 6 (54.5%) | 7 (36.8%) | 27 (28.7%) | 0.374 |
| GOJ | 3 (27.3%) | 6 (31.6%) | 26 (27.7%) |  |
| Gastric | 2 (18.2%) | 6 (31.6%) | 41 (43.6%) |  |
| **Metastasis present** |  |  |  |  |
| Metastasis | 7 (63.6%) | 14 (73.7%) | 64 (68.1%) | 0.833 |
| No metastasis | 4 (36.4%) | 5 (26.3%) | 30 (31.9%) |  |
| **DDIR classification** |  |  |  |  |
| DDIR Negative | 11 (100%) | 16 (84.2%) | 72 (76.6%) | 0.164 |
| DDIR Positive | 0 (0%) | 3 (15.8%) | 22 (23.4%) |  |
| **DDIR Score** |  |  |  |  |
| Mean (SD) | 0.172 (0.104) | 0.259 (0.187) | 0.313 (0.202) | **<0.001** |
| Median (Min, Max) | 0.147(0.0503, 0.363) | 0.257 (-0.0222, 0.523) | 0.286 (-0.0886, 0.860) |  |

**Supplementary Table 17.** EGFR FISH results according to DDIR status in the GO2 GOA population.

|  | **DDIR Negative (n=99)** | **DDIR Positive (n=25)** | **p-value** |
| --- | --- | --- | --- |
| **EGFR Fish Result** |  |  |  |
| Amplified (FISH positive) | 11 (11.1%) | 0 (0%) | 0.415 |
| High polysomy (FISH positive) | 16 (16.2%) | 3 (12.0%) |  |
| High trisomy (FISH negative) | 2 (2.0%) | 1 (4.0%) |  |
| Low polysomy (FISH negative) | 17 (17.2%) | 7 (28.0%) |  |
| Low trisomy (FISH negative) | 25 (25.3%) | 8 (32.0%) |  |
| Disomy (FISH negative) | 28 (28.3%) | 6 (24.0%) |  |

**Supplementary Figure S25.** MCP analysis of cytotoxic T-lymphocytes according to EGFR FISH results in the GO2 adenocarcinoma population. EGFR amplification was associated with lower abundance of cytotoxic T-lymphocytes. HP – high polysomy.

**
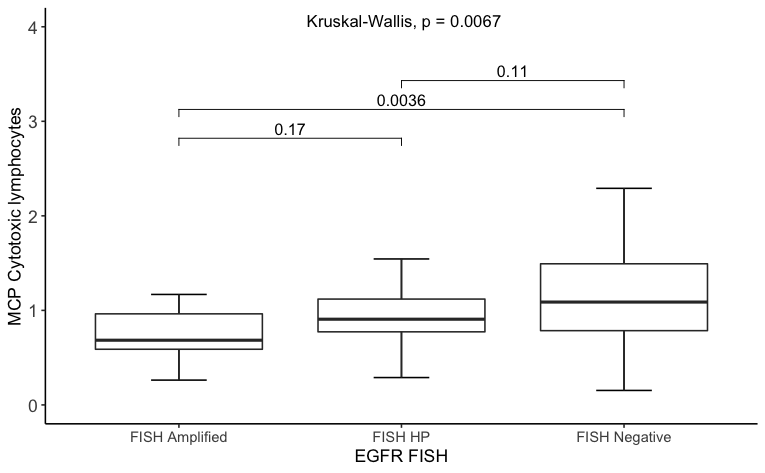
**

**Supplementary Figure S26.** MCP immune cell tumour microenvironment populations according to *EGFR* FISH results in the GO2 adenocarcinoma population. HP – high polysomy.

**Supplementary Table 18.** Demographics of the Grampian TMA population included in QuPath and PD-L1 analysis.

|  | **FISH Negative (N=93)** | **FISH Positive (N=31)** | **TOTAL (N=124)** | **P-value** |
| --- | --- | --- | --- | --- |
| **Age at Diagnosis** |  |  |  |  |
| Mean (SD) | 64.8 (10.4) | 63.8 (11.2) | 64.6 (10.6) | <0.001 |
| Median (Min, Max) | 64.0 (25.0, 88.0) | 66.0 (43.0, 84.0) | 66.0 (25.0, 88.0) |  |
| **Age Group** |  |  |  |  |
| <65 | 48 (52.2%) | 13 (41.9%) | 61 (49.6%) | 0.615 |
| >65 | 44 (47.8%) | 18 (58.1%) | 62 (50.4%) |  |
| **Sex** |  |  |  |  |
| Female | 27 (29.0%) | 9 (29.0%) | 36 (29.0%) | 1 |
| Male | 66 (71.0%) | 22 (71.0%) | 88 (71.0%) |  |
| **Primary Site** |  |  |  |  |
| Stomach | 33 (35.5%) | 13 (41.9%) | 46 (37.1%) | 0.813 |
| Oesophagus | 60 (64.5%) | 18 (58.1%) | 78 (62.9%) |  |
| **Stage** |  |  |  |  |
| Stage 1 | 20 (21.5%) | 7 (22.6%) | 27 (21.8%) | 0.582 |
| Stage 2 | 37 (39.8%) | 8 (25.8%) | 45 (36.3%) |  |
| Stage 3 | 34 (36.6%) | 13 (41.9%) | 47 (37.9%) |  |
| Stage 4 | 2 (2.2%) | 3 (9.7%) | 5 (4.0%) |  |

**Supplementary Table 19.** QuPath results from the Grampian TMA of patients with oesophageal and gastroesophageal junctional adenocarcinoma. Immune hot defined as the top 25% cell count density on immunohistochemistry. HP – high polysomy.

|  | **FISH Negative** | **FISH Positive** | |  |
| --- | --- | --- | --- | --- |
|  | **Negative (N=93)** | **Amplified (N=9)** | **HP (N=22)** | **P-value** |
| **CD8 Group** |  |  |  |  |
| Immune cold | 55 (74.3%) | 9 (100.0%) | 15 (71.4%) | 0.203 |
| Immune hot (top 25%) | 19 (25.7%) | 0 (0%) | 6 (28.6%) |  |
| **CD4 Group** |  |  |  |  |
| Immune cold | 63 (78.8%) | 8 (88.9%) | 12 (54.5%) | 0.041 |
| Immune hot (top 25%) | 17 (21.3%) | 1 (11.1%) | 10 (45.5%) |  |
| **FOXP3 Group** |  |  |  |  |
| Immune cold | 59 (78.7%) | 6 (66.7%) | 15 (68.2%) | 0.491 |
| Immune hot (top 25%) | 16 (21.3%) | 3 (33.3%) | 7 (31.8%) |  |

**Supplementary Table 20.** Distribution of PD-L1 combined positivity score (CPS) according to *EGFR* FISH result.

|  | **Negative (N=72)** | **High Polysomy (N=22)** | **Amplified (N=9)** | **Whole cohort (N=103)** | **P-value** |
| --- | --- | --- | --- | --- | --- |
| **PD-L1 CPS** |  |  |  |  |  |
| <5% | 64 (88.9%) | 18 (81.8%) | 9 (100%) | 91 (88.3%) | 0.548 |
| >5% | 8 (11.1%) | 4 (18.2%) | 0 (0%) | 12 (11.7%) |  |
| **PD- L1 CPS** |  |  |  |  |  |
| <1% | 60 (83.3%) | 17 (77.3%) | 9 (100%) | 86 (83.5%) | 0.494 |
| >1% | 12 (16.7%) | 5 (22.7%) | 0 (0%) | 17 (16.5%) |  |

**Supplementary Table 21.** Immune cell infiltrate (IHC) on QuPath, classified as immune hot and immune cold, according to PD-L1 combined positivity score (CPS) in the Grampian TMA cohort. Immune hot defined as top 25% of immune cell expression within cohort.

|  | **<5% (N=61)** | **5-10% (N=15)** | **10-49% (N=18)** | **>50% (N=14)** | **P-value** |
| --- | --- | --- | --- | --- | --- |
| **CD8** |  |  |  |  |  |
| Immune cold | 47 (85.5%) | 13 (86.7%) | 13 (72.2%) | 4 (28.6%) | <0.001 |
| Immune hot | 8 (14.5%) | 2 (13.3%) | 5 (27.8%) | 10 (71.4%) |  |
| **CD4** |  |  |  |  |  |
| Immune cold | 54 (88.5%) | 9 (60.0%) | 15 (83.3%) | 2 (14.3%) | <0.001 |
| Immune hot | 7 (11.5%) | 6 (40.0%) | 3 (16.7%) | 12 (85.7%) |  |
| **FOXP3** |  |  |  |  |  |
| Immune cold | 54 (88.5%) | 12 (80.0%) | 11 (61.1%) | 5 (35.7%) | <0.001 |
| Immune hot | 7 (11.5%) | 3 (20.0%) | 7 (38.9%) | 9 (64.3%) |  |

**Supplementary References**

1. Hall, P.S., et al., *Efficacy of Reduced-Intensity Chemotherapy With Oxaliplatin and Capecitabine on Quality of Life and Cancer Control Among Older and Frail Patients With Advanced Gastroesophageal Cancer: The GO2 Phase 3 Randomized Clinical Trial.* JAMA Oncology, 2021.

2. Dahle-Smith, Å., et al., *Epidermal Growth Factor (EGFR) copy number aberrations in esophageal and gastro-esophageal junctional carcinoma.* Mol Cytogenet, 2015. **8**: p. 78.

3. Craig, S.G., et al., *Immune status is prognostic for poor survival in colorectal cancer patients and is associated with tumour hypoxia.* British Journal of Cancer, 2020. **123**(8): p. 1280-1288.

4. Bankhead, P., et al., *QuPath: Open source software for digital pathology image analysis.* Scientific Reports, 2017. **7**(1): p. 16878.

5. Turkington, R.C., et al., *Immune activation by DNA damage predicts response to chemotherapy and survival in oesophageal adenocarcinoma.* Gut, 2019: p. gutjnl-2018-317624.

6. Turkington, R.C., et al., *Association of a DNA damage response deficiency (DDRD) assay with prognosis in resected esophageal and gastric adenocarcinoma.* Journal of Clinical Oncology, 2017. **35**(15_suppl): p. 4026-4026.

7. Mulligan, J.M., et al., *Identification and validation of an anthracycline/cyclophosphamide-based chemotherapy response assay in breast cancer.* J Natl Cancer Inst, 2014. **106**(1): p. djt335.

8. Hopewell, E.L., et al., *Lung tumor NF-κB signaling promotes T cell-mediated immune surveillance.* J Clin Invest, 2013. **123**(6): p. 2509-22.

9. Ayers, M., et al., *IFN-γ-related mRNA profile predicts clinical response to PD-1 blockade.* J Clin Invest, 2017. **127**(8): p. 2930-2940.

10. Beck, A.H., et al., *The macrophage colony-stimulating factor 1 response signature in breast carcinoma.* Clin Cancer Res, 2009. **15**(3): p. 778-87.

11. Ji, R.R., et al., *An immune-active tumor microenvironment favors clinical response to ipilimumab.* Cancer Immunol Immunother, 2012. **61**(7): p. 1019-31.
